# Directionally Locked Heteroepitaxy with a Structurally Modulated van der Waals Material

**DOI:** 10.1021/acsnano.6c04146

**Published:** 2026-06-23

**Authors:** Nitish Mathur, Guangming Cheng, Francesc Ballester, Gabrielle Carrel, Vincent M. Plisson, Fang Yuan, Jiangchang Zheng, Caiyun Chen, Scott B. Lee, Ratnadwip Singha, Sudipta Chatterjee, Kenji Watanabe, Takashi Taniguchi, Kenneth S. Burch, Berthold Jäck, Ion Errea, Maia G. Vergniory, Nan Yao, Sanfeng Wu, Leslie M. Schoop

**Affiliations:** † Department of Chemistry, 6740 Princeton University, Princeton, New Jersey 08544, United States; ‡ Princeton Materials Institute, Princeton, New Jersey 08544, United States; § 226245 Donostia International Physics Center, 20018 Donostia-San Sebastián, San Sebastián 20018, Spain; ∥ Department of Applied Physics, University of the Basque Country (UPV/EHU), 20018 Donostia-San Sebastián, San Sebastián 20018, Spain; ⊥ Department of Physics, Boston College, Chestnut Hill, Massachusetts 02467, United States; # Department of Physics, 540065 The Hong Kong University of Science and Technology, Clear Water Bay, Kowloon 999077, Hong Kong; ¶ Department of Physics, 28678 Indian Institute of Technology Guwahati, Assam 781039, India; ∇ Research Center for Electronic and Optical Materials, 52747 National Institute for Materials Science, 1-1 Namiki, Tsukuba 305-0044, Japan; ○ Research Center for Materials Nanoarchitectonics, National Institute for Materials Science, 1-1 Namiki, Tsukuba 305-0044, Japan; ⧫ Centro de Física de Materiales (CSIC-UPV/EHU), 20018 Donostia-San Sebastián, San Sebastián 20018, Spain; †† Département de Physique et Institut Quantique, Université de Sherbrooke, Sherbrooke J1K 2R1, Québec, Canada; ‡‡ Department of Physics, Princeton University, Princeton, New Jersey 08544, United States

**Keywords:** van der Waals materials, epitaxy, structural modulation, scanning transmission electron microscopy, lattice instability

## Abstract

Precise orientation of symmetry-mismatched epilayers on van der Waals (vdW) substrates via heteroepitaxy has commonly been achieved through surface treatment processes to accommodate weak interlayer registry and bonding strength, thereby limiting the range of material combinations for heterostructure design. In this study, we investigate the influence of lattice instabilities in a structurally modulated vdW TaCo_2_Te_2_ substrate on the growth and alignment of a symmetry-mismatched bulk Co_*x*_Te_*y*_ epilayer using in situ heating in a transmission electron microscope (TEM). We show that a Peierls-like lattice instability occurs in TaCo_2_Te_2_ at a transition temperature of ∼523 K, which was corroborated by phonon calculations. Postheat-treated samples reveal a thermally induced surface diffusion process and the dominant lateral growth of the Co_*x*_Te_*y*_ epilayer on the TaCo_2_Te_2_ vdW layers, as observed in cross-sectional TEM images. Temperature-dependent selected area electron diffraction (SAED) patterns reveal that the quasi-vdW Co_*x*_Te_*y*_/TaCo_2_Te_2_ heterointerface acquires directional locking by aligning larger interlayer lattice mismatch along the lattice instability axis of TaCo_2_Te_2_, while preserving a strong lattice matching along the orthogonal direction. This heterostructure exhibits precise interlayer registry with one-dimensional lattice incommensuration along the lattice instability axis, resulting from structural distortion to accommodate lattice-mismatch strain. Moreover, the interfacial reconstruction of TaCo_2_Te_2_ back to the distorted phase stabilizes the lattice-locking of the quasi-vdW heterointerface at elevated temperatures. These findings encourage the expansion of material diversity for designing and predicting multidimensional heterostructures by leveraging lattice instabilities to guide epitaxy.

## Graphical abstract

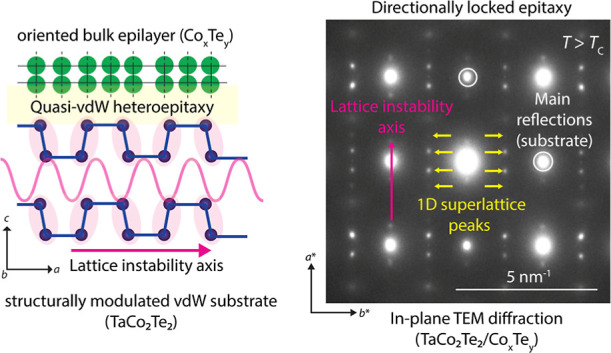

## Introduction

Developing methods to interface van der Waals (vdW) materials with three-dimensional (3D) bulk materials via heteroepitaxy is of strong interest, which supports multijunction device architectures for future downscaled electronics.^1,2^ Wafer-scale growth of vdW heterostructures has been successfully achieved via heteroepitaxy.^3,4^ Here, the lack of strong chemical bonds at the surface of vdW materials successfully accommodates interfacial strain and prevents dislocations caused by the lattice mismatch between interfacing materials.^5^ Similarly, the growth of 3D bulk materials on a vdW substrate (or vice versa) occurs via quasi-vdW (QvdW) epitaxy. The resulting “hybrid bonding” at the 2D/3D interface consists of weak vdW forces and dangling-bond or electrostatic interactions.^6,7^ In previous studies, well-aligned interfaces have been formed in heterostructures via QvdW epitaxy between common 2D materials, such as transition-metal dichalcogenides (TMDs), and 3D bulk metals or semiconductor compounds.^5,6,8–12^ It makes intuitive sense to interface materials with matching interfacial symmetry to achieve precise epitaxial conditions without rotational misalignment between the epilayer and the substrate.^13–15^ Previous studies have shown that the strength of QvdW interfacial interactions in symmetry-mismatched heterostructures dictates whether preferential alignment or rotational disorder relaxes the high-energy heterointerface.^16,17^ Generally, growth dynamics have been controlled by presurface treatment of the vdW substrate to promote epilayer–substrate interactions. It involves processes such as creating step edges, plasma treatment, or introducing an amorphous buffer seed layer to achieve lattice-locked heteroepitaxy.^18–21^ Strong epilayer crystallinity and interlayer registry during heteroepitaxy are critical for wafer-level material growth.

Beyond weak interlayer vdW forces, strain from interfacing multidimensional (2D/3D) materials can be further accommodated by the inherent flexibility of intralayer atomic bonding.^22^ Considering structural flexibility as the key criterion, structurally modulated vdW materials could be suitable growth substrates for accommodating symmetry-mismatched heterointerfaces. Modulated structures are a distinct class of materials characterized by atomic positions that systematically deviate from the ideal periodic lattice in one or more crystallographic directions.^23–25^ The deviation from the ideal lattice could be either commensurate, incommensurate, or both with respect to the underlying lattice. These are extensively studied for charge density wave (CDW) phases in vdW materials.^26–29^ Above the transition temperature (*T*_C_), the low-symmetry modulated structure becomes thermally unstable and deforms into a more stable high-symmetry unmodulated structure. This structural phase transformation is characterized by lattice instability. More commonly, these lattice instabilities in a structurally modulated material near *T*_C_ transform into dynamical lattice fluctuations that can persist as short-range order (SRO) even above *T*_C_.^30^ Previous studies have shown that the short-range lattice distortions in the vicinity of crystalline defects, such as edge dislocations, can influence the growth mechanism and the resulting orientation of the epilayer.^31,32^ Similarly, lattice fluctuations are intrinsic to a modulated material that exhibits both anisotropy and dynamical features of soft phonon modes. The question remains whether this inherent flexibility in vdW-modulated-based heterostructures can enable precise orientation during epilayer growth, or whether thermal and interfacial strain effects hinder orientation lock-in. Additionally, the influence of these lattice instabilities on heteroepitaxy is still poorly understood, as this phenomenon is rarely observed above room temperature in vdW-modulated structures where nucleation and growth occur.^33–36^

In this work, we introduce vdW TaCo_2_Te_2_ as a growth substrate that exhibits a modulated structure and lattice instability above room temperature. We chose TaCo_2_Te_2_ because it exhibits a characteristic Peierls-like distortion, allowing us to examine how anisotropic phonon instability affects epilayer growth. We also compare our results with the isostructural TaNi_2_Te_2_, an undistorted version of TaCo_2_Te_2_, to highlight differences in structural evolution during heteroepitaxy. We analyze the growth of 3D Co_*x*_Te_*y*_ epilayer on TaCo_2_Te_2_ nanoflakes above *T*_C_ using in situ heating in a scanning/transmission electron microscope (S/TEM). The analysis reveals that the large lattice-mismatch axis of the heterointerface aligns with the lattice instability axis of TaCo_2_Te_2_, while the in-plane orthogonal axis shows strong lattice matching. We then compare our results with the isostructural undistorted TaNi_2_Te_2_ system and find that the epilayer growth shows rotational misalignments in the interlayer registry. We show that interactions between anisotropic lattice instabilities and QvdW bonding stabilize a one-dimensional (1D) structural modulation at the Co_*x*_Te_*y*_/TaCo_2_Te_2_ heterointerface and a dominant lateral epilayer growth, thereby facilitating directionally locked QvdW epitaxy. Therefore, Ta­(Co,Ni)_2_Te_2_ systems allow examination of how lattice instability guides heteroepitaxial growth.

## Results and Discussion

### Structurally Modulated Lattice of TaCo_2_Te_2_

TaCo_2_Te_2_ is a metallic vdW material with an orthorhombic structure (space group 62, more details of structural refinement in Tables S1–S4) and a sextuple-atomic monolayer^37,38^ with a structural modulation along the *a*-axis (Figure 1a) at room temperature (RT). The sample must be heated above RT to obtain a high-symmetry undistorted structure with the unit cell halved along the *a*-axis. The TaCo_2_Te_2_ undistorted structure is isostructural with TaNi_2_Te_2_ (Figure S1). Using chemical vapor transport (CVT), we synthesize single crystals of TaCo_2_Te_2_ (see Methods, Figure S2, and Table S5) and find that ultrathin TaCo_2_Te_2_ nanoflakes from bulk crystals can be easily acquired via mechanical exfoliation and do not degrade under ambient conditions (Figure S3). We prepare TEM samples by both transferring exfoliated nanoflakes onto a TEM grid and by cutting a thin lamella with a focused ion beam (FIB). Cross-sectional scanning transmission electron microscopy (STEM) images (Figure 1b,d) elucidate the transition from the RT distorted structure to the undistorted high temperature structure, as shown in Figure 1c,d. We index all TaCo_2_Te_2_ SAED images with respect to the RT-distorted phase. The main reflections of the TaCo_2_Te_2_ RT phase also comprise peaks from the modulated structure (Figure 1e), exhibiting commensurate unit cell wavevectors (*q*_c_) indexed to the (110) family of planes. We often observe forbidden reflections arising from deviations in the orthorhombic unit cell, which may be caused by residual strains during nanoflake preparation or by strain release via stacking faults during sample heating (Figure S4).^33,39^

**Figure 1:**
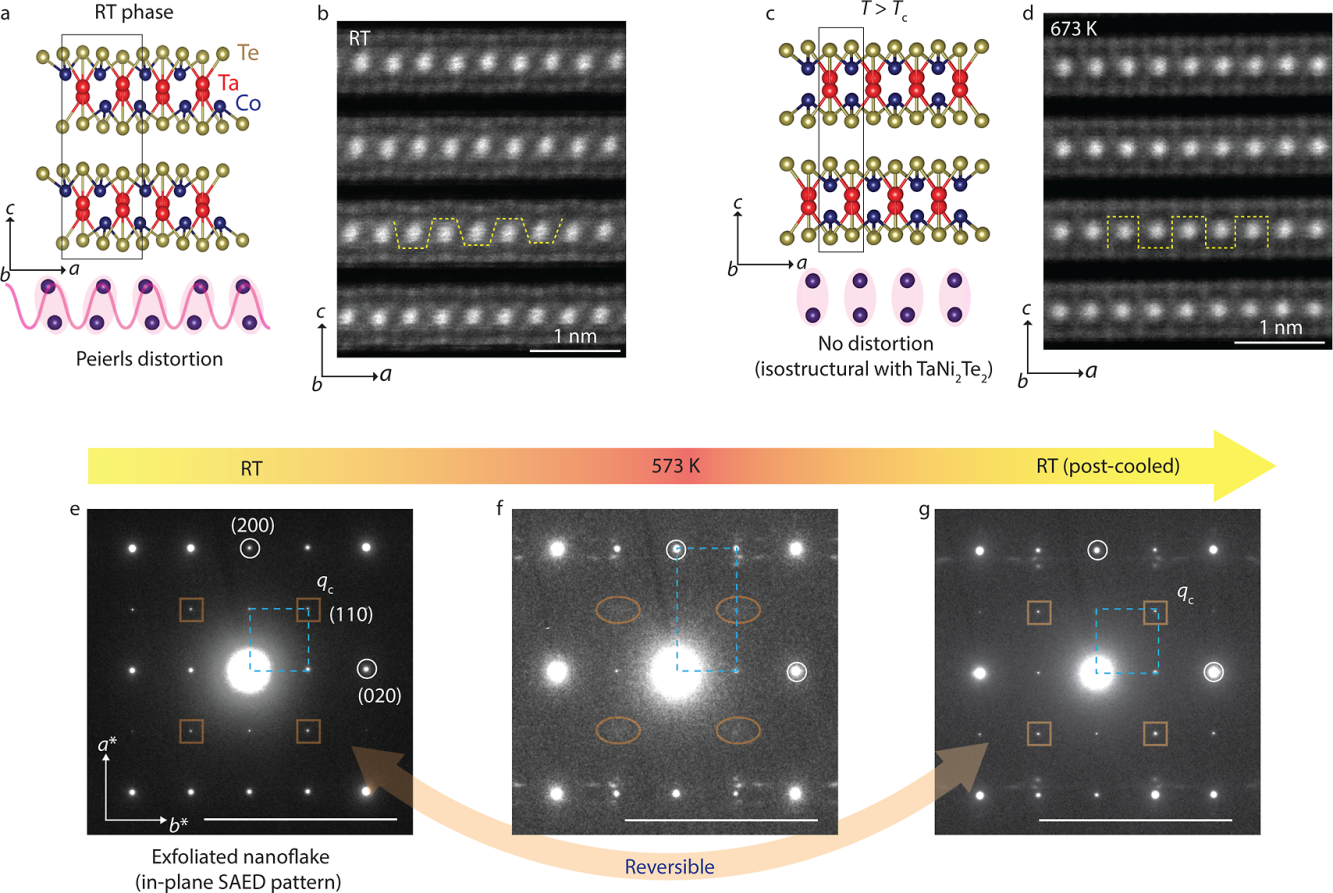
Structurally modulated TaCo_2_Te_2_. (a–d) Crystal structure of the room temperature (RT) phase of TaCo_2_Te_2_ with structural modulation, known as Peierls distortion, along the crystallographic *a*-axis and crystal structure of the undistorted phase above *T*_C_. Black boxes in panels a and c represent unit cells of both phases. Cross-sectional STEM images of the *ac*-plane showing the transformation from the RT distorted structure to the undistorted structure. Dotted yellow lines highlight Co atomic chains matching corresponding crystal structures in panels a,c (e–g) SAED images revealing a reversible structural transition in an exfoliated TaCo_2_Te_2_ nanoflake. Diffraction peaks associated with the Peierls distortion, marked with orange boxes­(ellipsoids), indicate the presence­(absence) of characteristic commensurate wavevectors (*q*_c_) when compared with the lattice parameter of the undistorted reciprocal unit cell marked in dashed blue boxes. Scale bar in panels (e)–(g) is 5 nm^–1^. All TaCo_2_Te_2_ SAED images are indexed with respect to the RT distorted structure.

We determine the temperature-dependent structural evolution of the distorted phase from in-plane SAED images of a TaCo_2_Te_2_ nanoflake. Here, we observe incoherent peaks at *q*_c_ above *T*_C_ as the structure loses its low-symmetry distorted phase (Figure 1e) and transitions to a more stable high-symmetry undistorted phase (Figure 1f). Subsequently, the reciprocal unit cell doubles along the a* direction, which is characteristic of the Peierls-like transition. We fully recover the TaCo_2_Te_2_ distorted phase after cooling from *T*_C_ (Figure 1g), showing a reversible structural transition. We also detect sharp peaks in differential scanning calorimetry (DSC) scans corresponding to the structural phase transition and obtain a more precise value of *T*_C_ ≈ 523 K (Figure S5). Scanning tunneling microscopy (STM) topography acquired at ∼4.2 K shows the atomically resolved lattice of the TaCo_2_Te_2_ (001) surface (Figure S6). Fourier transformed (FFT) image of the topography reveals modulation peaks in addition to Bragg peaks, as in the SAED image at RT. We determine the corresponding real-space modulation (*a*_stripe_ ≈ 0.67 ± 0.01 nm) from the stripe-like pattern by performing an inverse FFT on peaks along the a* direction. This value is close to the RT lattice parameter (*a* ≈ 0.66 nm) of the TaCo_2_Te_2_ distorted structure. Combining the results of TEM diffraction and STM images, we conclude that the modulated structure of TaCo_2_Te_2_ is stable over a wide temperature range.

### Lattice Instabilities in TaCo_2_Te_2_

To understand lattice instabilities during the structural transition, phonon-dispersion calculations for the undistorted structure of TaCo_2_Te_2_ at 0 K reveal pronounced imaginary frequencies across multiple high-symmetry points in the Brillouin zone (BZ) (Figure 2a,b).^40^ The phonon spectra show instabilities at the *X*, *S*, *R*, and *U* points, which lie along the *k*_*x*_ direction, but also have components along the *k*_*y*_ and *k*_*z*_ directions. However, we found no instabilities at BZ points without a *k*_*x*_ component. This suggests that the unstable modes lie only along the *k*_*x*_ direction and coincide with the Peierls distortion axis in TaCo_2_Te_2_. We found that Co atoms contribute significantly more to this phonon instability than Ta and Te atoms (Figure S7). We conduct Raman measurements on an exfoliated TaCo_2_Te_2_ nanoflake (Figure S8) within a glovebox^41^ at various temperatures to determine changes in collective phonon modes during the structural phase transition. In Figure 2c, we show Raman spectra from RT to 600 K, with each temperature offset for clarity. The RT spectrum consists of a cluster of 4 modes from 90 cm^–1^ to 165 cm^–1^ with two additional weak modes between 250 cm^–1^ and 300 cm^–1^. There are two particularly interesting aspects of the temperature-dependent results that we focus on: the sudden change in the spectrum near *T*_C_ and the evolution of the P3 and P5 modes over the whole temperature range. While the spectra evolve continuously with increasing temperature, a significant change in the overall spectrum occurs around 550 K, marked by the appearance of a double peak near the P3 mode at 135 cm^–1^. At the same time, the weak P4 mode appears to vanish. These two features become even more significant at the highest temperatures above *T*_C_. Beyond the differences in the overall spectra above and below *T*_C_, our measurements also reveal an unusual shift in the Raman peaks of the P3 mode. The energy shifts of both these modes are plotted in Figure 2d. Typically, phonons shift to lower energies as temperature increases due to lattice expansion. This is precisely how the P5 mode behaves. It gradually softens through P3 mode as it transitions to higher energy levels with increasing temperature. This behavior is indicative of additional higher-order anharmonic terms,^42^ most likely due to some avoided band crossings or structural transition above *T*_C_. In situ heating of an exfoliated TaCo_2_Te_2_ nanoflake above *T*_C_ results in a structural transition to the undistorted phase. The persistence of SRO above *T*_C_ is evident in the SAED image, which reveals diffuse scattering peaks at *q*_c_ (Figure 3a). We determine the preferred orientation of incoherent structural domains above *T*_C_ by measuring the spread of a representative SRO diffuse peak at the full-width half-maximum (fwhm) intensity along the a* and b* directions (Figure S9). The SRO correlation length is longer along the *a*-axis (≈2.12 nm) compared to the *b*-axis (≈1.35 nm). Hence, the anisotropic nature of lattice fluctuations is evident in TaCo_2_Te_2_ above *T*_C_.

**Figure 2:**
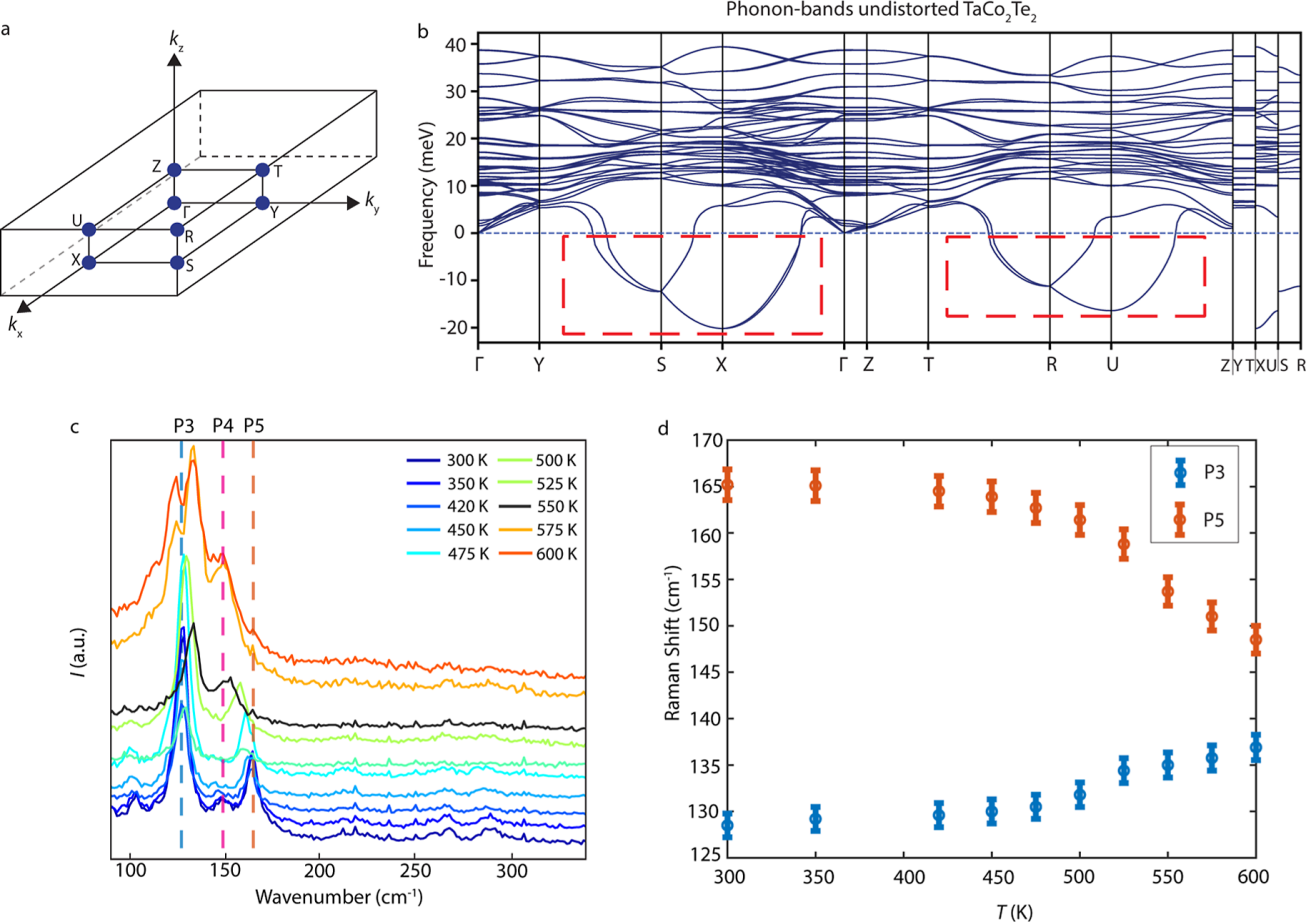
Lattice instabilities in TaCo_2_Te_2_. (a) 3D Brillouin zone of the undistorted TaCo_2_Te_2_ structure used for phonon-dispersion calculations. (b) Phonon band calculations of the undistorted TaCo_2_Te_2_ above *T*_C_. Dashed red boxes highlight imaginary phonon bands expanding over a wide region of the Brillouin zone. (c) Raman spectrum collected on a TaCo_2_Te_2_ nanoflake at various temperatures above RT. Raman modes P3, P4, and P5 are marked with blue, pink, and orange dashed lines, respectively. (d) Corresponding temperature dependence of P3 and P5 Raman peak shifts.

**Figure 3:**
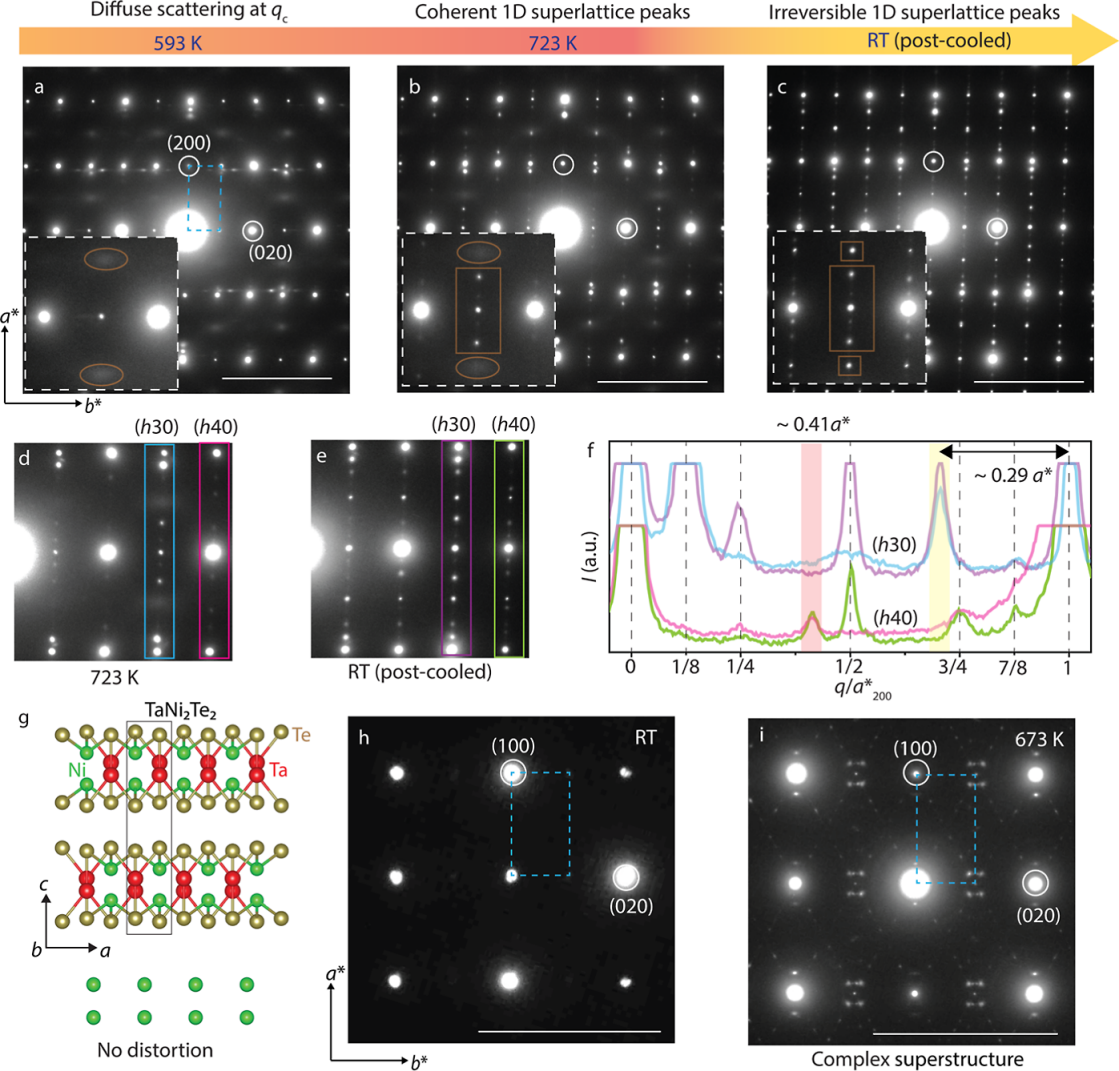
Emergence of superlattice structure observed using in situ heating above *T*_C_. Structure evolution of the TaCo_2_Te_2_ structure above *T*_C_ is shown in the in-plane SAED image at (a) 593 K, (b) 723 K, and (c) RT (postcooled). Insets highlight the corresponding diffuse peaks at *q*_c_ associated with SRO, marked by orange ellipsoids, and the coherent 1D superlattice peaks around the main reflections, marked by an orange rectangular box. (d,e) Zoomed images of SAED patterns at 723 K and RT (postcooled). (f) Representative intensity line scans along the a* direction for (*h*30) and (*h*40) planes in the reciprocal space, as shown in panels (d) and (e). The colors of rectangular boxes marked in panels (d) and (e) correspond to the colors of trace lines in the intensity plot. (g) Schematic of TaNi_2_Te_2_ shows the RT phase, isostructural to the undistorted TaCo_2_Te_2_ phase. SAED patterns show the temperature-dependent structure evolution from the (h) RT TaNi_2_Te_2_ phase to a (i) complex superstructure at 673 K. Scale bar in panels a–c, h, and i is 5 nm^–1^. SAED images in panels h and i are indexed with the TaNi_2_Te_2_ RT phase.

### Emergence of Superlattice Peaks in TaCo_2_Te_2_ and TaNi_2_Te_2_

Above *T*_C_, we also observe the emergence of the new coherent peaks with increasing temperature in in-plane SAED images of the TaCo_2_Te_2_ exfoliated nanoflake. This manifests as superlattice peaks only along the a* direction (Figure 3b). The persistence of 1D superlattice peaks in the SAED image even after the sample has cooled and rested at RT for an extended time indicates an irreversible phase transition (Figure 3c). However, these irreversible superlattice peaks are absent in the SAED image of the postcooled FIB sample after annealing, where we milled a thickness of 5–10 nm from both the top and bottom (Figure S10a–c). Additionally, superlattice peaks emerge even when the lamella is heated under beam-blanked conditions in a TEM holder, suggesting no effects of the electron beam on structural evolution above *T*_c_ (Figure S10d,e). Therefore, a new crystalline layer with a different crystal structure forms on the surface of TaCo_2_Te_2_ nanoflakes when heated above *T*_C_. We compare the representative superlattice peaks along the a* direction in SAED images (Figure 3d,e) collected at 723 K and at RT (postcooled). The analysis of SAED images reveals characteristic superlattice peaks appearing at wavevectors *q*_1_ ≈ 0.12 a*_200_ and *q*_2_ ≈ 0.29 a*_200_ corresponding (*hk*0) main reflections (where *k* is an odd integer), while their corresponding higher-order wavevectors ≈0.24 a*_200_ (2*q*_1_) and ≈0.41 a*_200_ (1–2*q*_2_) appear for (*hk*0) main reflections (where *k* is an even integer or 0) as shown in Figure 3f. We also observe weakly coherent peaks in SAED patterns at incommensurate wavevectors that do not orient along the a* direction (Figures 3a and S11). These weakly coherent peaks disappear completely as we increase the temperature above *T*_C,_ indicating that they are linked to kinetic trapping during surface growth. To verify the robustness and alignment of superlattice peaks in TaCo_2_Te_2_, we then conduct in situ heating TEM experiments on an in-plane FIB-prepared lamella, as this sample preparation method differs from that used for exfoliated/transferred TaCo_2_Te_2_ nanoflakes (Figures S12 and S13). Indeed, acquired SAED images at increasing temperatures above *T*_C_ show similar 1D superlattice peaks with wavevectors (*q*_1_, *q*_2_) along the a* direction (Figure 3d,e). Note that *q*_1_ and *q*_2_ are unaffected by decreasing temperature below *T*_C_, where the TaCo_2_Te_2_ Peierls distortion occurs. This suggests that the superlattice peaks (*q*_1_, *q*_2_) are not associated with new TaCo_2_Te_2_ lattice instabilities. To confirm this further, we perform in situ heating on exfoliated TaNi_2_Te_2_ nanoflakes (isostructural with the undistorted phases of TaCo_2_Te_2_), which do not show lattice instabilities like TaCo_2_Te_2_ (Figure 3g,h).^37^ We still observe the emergence of a complex superstructure in the SAED pattern collected at 673 K in TaNi_2_Te_2_ (Figure 3i). Despite the isostructural nature of TaNi_2_Te_2_ and undistorted TaCo_2_Te_2_, the superlattice structure differs significantly between them. These observations suggest that the crystalline symmetry of the underlying substrate is not the sole factor determining the interlayer registry of the superstructure.

### Growth of Surface Layer on TaCo_2_Te_2_

We examine the thermally induced surface layer (SL) growth using FIB-prepared TaCo_2_Te_2_ cross-sectional TEM samples by sandwiching a TaCo_2_Te_2_ nanoflake between a hexagonal boron nitride (hBN) nanoflake and an amorphous carbon layer (see Methods and Figure S14). An atomic-resolution annular dark-field (ADF)-STEM image (Figure 4a) near the surface of postcooled cross-sectional TEM samples (after heating in situ at 753 K for 6 h and then cooling back down to RT) reveals the formation of a 2–3 nm thick new SL. The new SL consists of an ordered SL (OSL) covered by an amorphous SL (ASL). TEM images show that SL uniformly extends laterally across the external surfaces and near the TEM sample’s side edges (Figure S15). The STEM image contrast decreases in the SL compared to the TaCo_2_Te_2_ vdW layers, which are typically proportional to the atomic number of the constituent elements and the total number of atoms in the atomic column. We used TEM-EDS elemental mapping (Figures 4b–f) to investigate this discrepancy, revealing phase segregation in the SL due to surface diffusion at elevated temperatures. This results in Ta atoms migrating to the ASL, whereas Co and Te remain in the OSL. Quantitative analysis using a line scan near the new SL reveals a sharp drop in the Ta relative atomic percentage (%) in the OSL (Figure 4g). At the same time, the Co % and Te % remain close to 40%, similar to the TaCo_2_Te_2_ vdW layers. Note that the image drift during the elemental mapping scans does not allow for an exact value of relative elemental composition shown in the line scan plot. Nevertheless, we determine that the Ta-deficient OSL accounts for the decrease in STEM intensity compared to TaCo_2_Te_2_ vdW layers.

**Figure 4:**
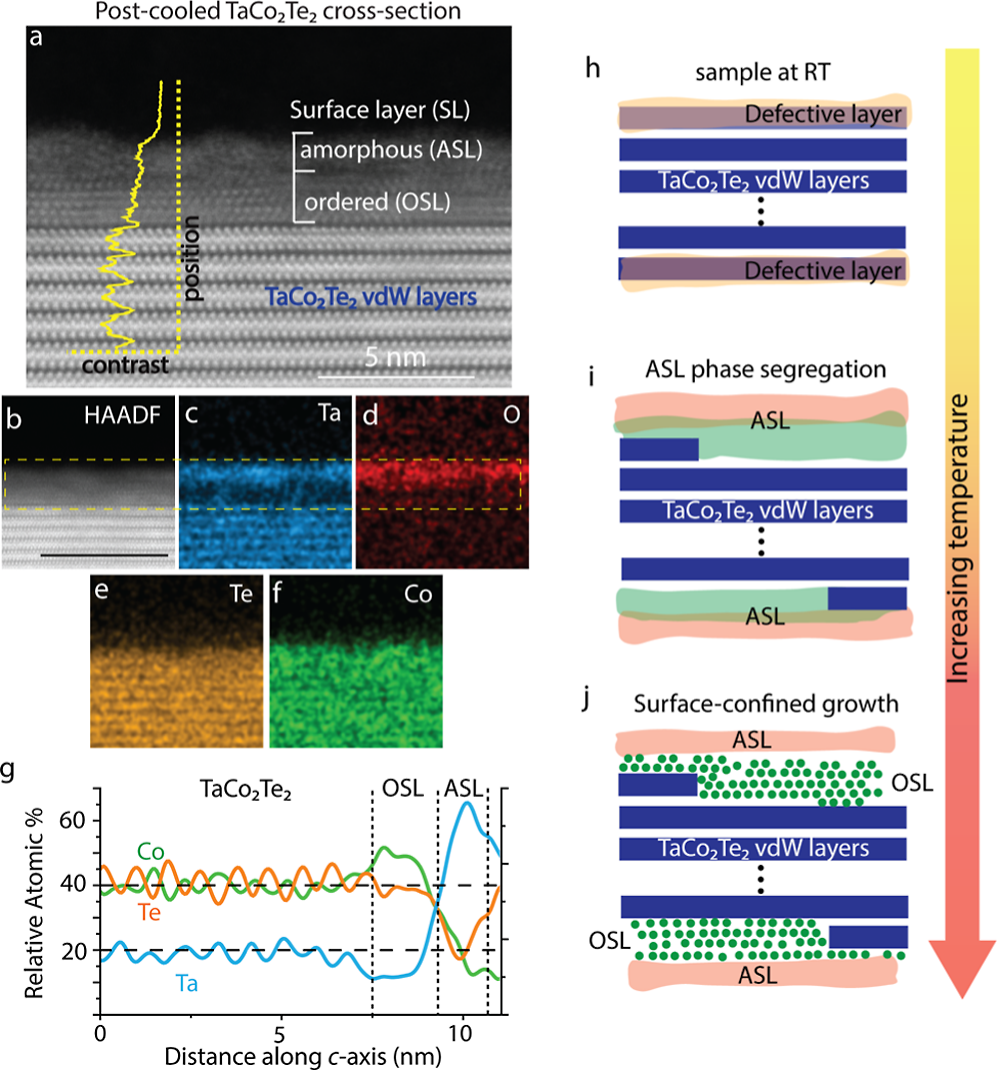
Growth of surface layer (SL) on TaCo_2_Te_2_vdW layers. (a) Cross-sectional ADF-STEM image of the postcooled TaCo_2_Te_2_ FIB-prepared sample showing a SL consisting of amorphous (ASL) and ordered (OSL) layers. The SL layer shows weaker STEM contrast than the TaCo_2_Te_2_ layers. (b–f) Elemental mapping of the corresponding cross-sectional HAADF image. The yellow dash box highlighting the Ta and O migration in the ASL layer. (g) Quantitative elemental line scan along the *c*-axis marked in a yellow dashed line in panel a. (h–j) Schematics illustrating the temperature-dependent structure evolution of SL on TaCo_2_Te_2_ vdW layers. Scale bar in panel (b) is 10 nm.

Based on these observations, we can elucidate the progression of SL growth in TaCo_2_Te_2_ at temperatures above *T*_C_, as shown in the illustration in Figures 4h–j. The combined effect of defects, such as vacancies and oxidation-induced amorphization, which are more likely to occur at the sample surface and edges, facilitates thermally induced surface-confined diffusion reactions. TEM samples prepared via high-energy FIB milling exhibit greater surface amorphization than those prepared by the nanoflake exfoliation/transfer method. We observe that the superlattice peaks emerge at a slightly lower temperature (about 50 K lower) than in exfoliated nanoflakes. Here, atoms diffuse more easily in the disordered phase to form SL postheating, indicating a lower thermal barrier for surface–diffusion reactions. TEM-EDS scans reveal that Ta % increases in the ASL layer, where O % is also significantly higher (Figure 4c,d). This is expected due to the higher O affinity for Ta.^43^ The resulting phase-segregated ASL sublayers are likely to form with high concentrations of Ta–O and Co–Te. Above *T*_C_, only Co–Te forms an OSL layer within the operating temperature range and forms a heterostructure with TaCo_2_Te_2_. Similar phase reconstructions have been reported in GeBi_2_Te_4_ following heat treatment, showing that atoms diffuse predominantly in-plane along the vdW gaps and surfaces.^44^ In TaCo_2_Te_2_, adatom diffusion within the lateral plane could be facilitated by anisotropic phonon softening above *T*_c_, thereby directly guiding the epilayer’s nucleation and growth. Furthermore, we observe that the Co_*x*_Te_*y*_ epilayer effectively wets the TaCo_2_Te_2_ substrate laterally before vertical growth. The growth style matches the Frank-van der Merwe (FV) model, characterized by layer-by-layer growth and an energetically favorable interface binding energy.^45,46^

### Directionally Locked Heteroepitaxy between the Epilayer and the TaCo_2_Te_2_ Substrate

Next, we determine the epitaxial conditions and atomic-level reorganization of the Co_*x*_Te_*y*_/TaCo_2_Te_2_ heterostructure. The Co_*x*_Te_*y*_ OSL interface with TaCo_2_Te_2_ at a quasi-vdW gap, as shown in the atomic resolution ADF-STEM image (Figure 5a). The crystal structures of Co_*x*_Te_*y*_ binary alloys typically adopt a hexagonal or trigonal symmetry, except for an orthorhombic symmetry (*P*nnm) previously reported for CoTe_2_.^47^ In the Co_*x*_Te_*y*_ OSL layer, the atomic column aligns along the [-2110] zone axis, where the Te atomic column exhibits brighter STEM intensity compared to the Co atomic column (Figure 5b). In the thinner regions near the edges of the TEM cross-sectional TEM sample, the interconnected chemically bonded Co and Te sublayers are clearly visible in the STEM image (Figures S16 and S17). This confirms that the 3D hexagonal CoTe non-vdW layered structure (hexagonal, space group no. 194) forms the OSL layer. While the exact Co_*x*_Te_*y*_ composition may vary across the surface layer, Co_*x*_Te_*y*_ still adopts a non-vdW hexagonal parent structure with minimal change in lattice parameters.^47^ We determine that the atomic columns of TaCo_2_Te_2_ in the cross-sectional STEM image closely align along the [120] zone axis (Figures 5c and S18). Hence, the resulting heterostructure aligns along their respective *c*-axes (Co_*x*_Te_*y*_ <0001>//TaCo_2_Te_2_ <001>) and should form a heterostructure with mismatched out-of-plane symmetry (Figure 5d,e).

**Figure 5:**
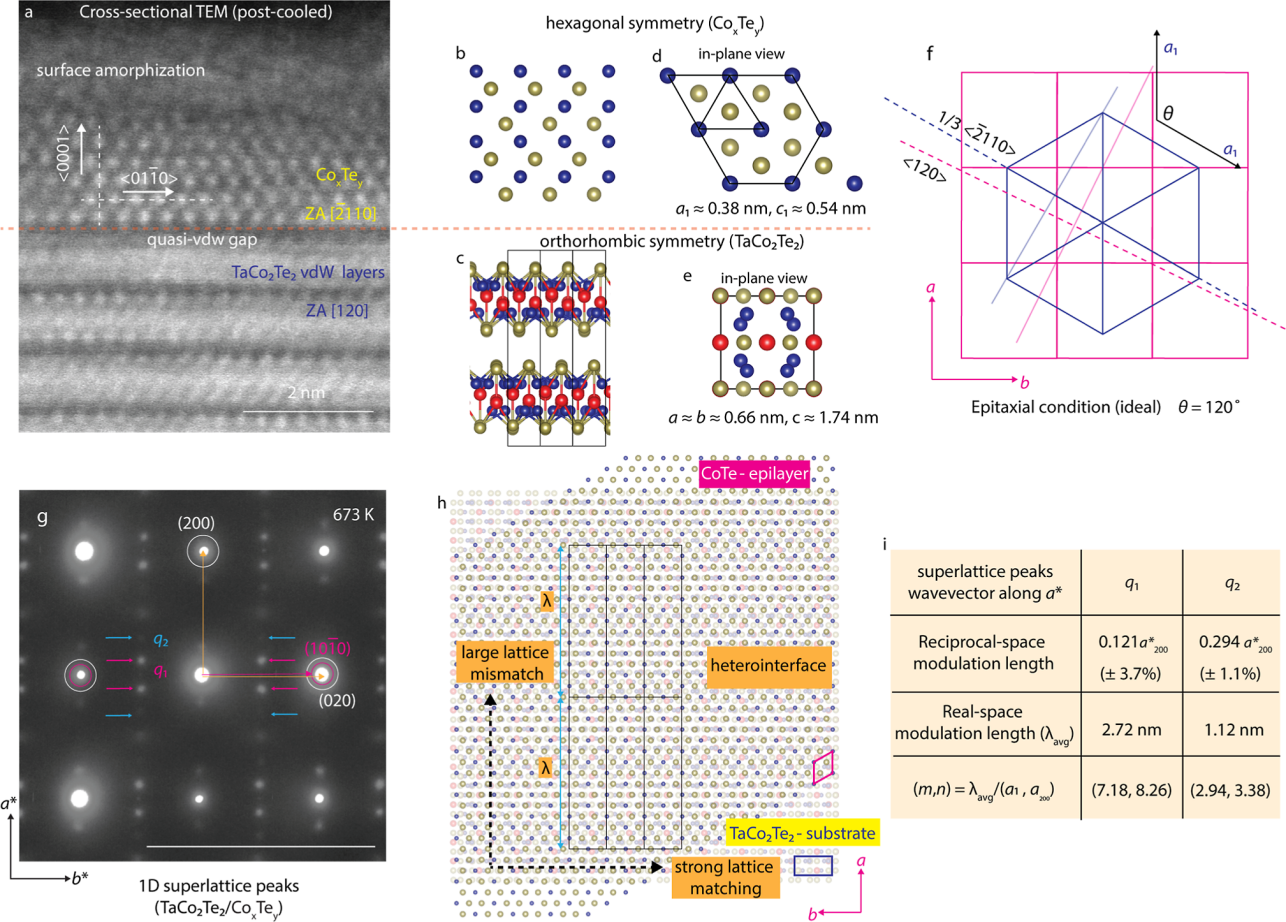
Structural analysis of QvdW Co_*x*_Te_*y*_/TaCo_2_Te_2_ directionally locked heteroepitaxy. (a) Cross-sectional atomic resolution ADF-STEM image of the Co_*x*_Te_*y*_/TaCo_2_Te_2_ near the surface of the lamella. (b,c) Crystal structures with layer stacking viewed along the cross-section of the CoTe/TaCo_2_Te_2_ heterostructure in panel a. In-plane view of the crystal structures, highlighting the mismatched symmetries of the (d) hexagonal CoTe and (e) orthorhombic TaCo_2_Te_2_. (f) Schematic showing the ideal epitaxial condition for the superlattice using a two-dimensional representation of the hexagonal CoTe and orthorhombic TaCo_2_Te_2_ in-plane lattice. Faded blue and pink lines highlight planes imaged in the cross-sectional STEM experiment shown in panel a. (g) In-plane SAED pattern of Co_*x*_Te_*y*_/TaCo_2_Te_2_ with 1D superlattice peaks. Pink circles highlight diffraction spots indexed to the hexagonal CoTe (0001) plane overlapping with TaCo_2_Te_2_ diffraction spots marked in white circles. (h) Schematic representation of the interlayer registry forming CoTe (hexagonal)/TaCo_2_Te_2_ (undistorted) heterostructure corresponding to the epitaxial condition in panel g. Black, pink, and dark blue boxes highlight superlattice, epilayer-CoTe, and substrate-TaCo_2_Te_2_ unit-cells (i) Comparison table of superstructure modulations along the *a*-axis from experimental data and the coincidence site lattice analysis. Scale bar in panel (g) is 5 nm^–1^.

The formation of 1D superlattice peaks along the lattice instability axis of the TaCo_2_Te_2_ unit cell shows that the QvdW heteroepitaxy of Co_*x*_Te_*y*_/TaCo_2_Te_2_ favors a specific epitaxial alignment. We show a schematic to illustrate the ideal epitaxial condition of the CoTe/TaCo_2_Te_2_ heterostructure from an in-plane view (Figure 5f). This proposed model matches the cross-sectional STEM image shown in Figure 5a, where the zone axes of hexagonal Co_*x*_Te_*y*_ 1/3<-2110> and TaCo_2_Te_2_ <120> are nearly aligned, with only minor deviations (ideally ≈3.4°). The superlattice along the b* direction is commensurate with the TaCo_2_Te_2_ substrate because *d*_010_ (CoTe) ≈ *d*_020_ (TaCo_2_Te_2_), where *d*_*hkl*_ is the interplanar distance. This perfect interlayer registry along b* is likely because the minimal theoretical lattice mismatch (Δ) between these lattice vectors is ≈2.2% and forms a lattice-locked axis of the heterostructure. In contrast, a significant lattice mismatch of approximately 17.5% exists along the a* direction, which coincides with the lattice instability axis of TaCo_2_Te_2_ (Figure S19). The average real space modulation length (λ_avg_) of the superlattice peaks acquired from the SAED images is ≈2.72 nm (for *q*_1_) and ≈1.12 nm (*q*_2_) (Figure 5g). We first check the possibility of a 1D Moiré pattern in the Co_*x*_Te_*y*_/TaCo_2_Te_2_ heterostructure to explain the superlattice wavevectors. The deviation between the calculated and experimental modulation lengths is quite large (≈24%). Further, we present a schematic of the overlaid atomic structure of orthorhombic TaCo_2_Te_2_-substrate (undistorted) and the hexagonal CoTe-epilayer heterointerface (Figure 5h) to determine the deviation from lattice commensurability along the *a*-axis using coincidence site lattice (CSL) analysis.^13^ The model states that the CSL sites occur at a periodic distance (λ) along the *a*-axis so that the lattice commensuration can be achieved if λ = *ma*_1_ = *na*, where *m* and *n* are integers, and *a*_1_ and *a* are the lattice parameters of CoTe and undistorted TaCo_2_Te_2_, respectively (see more details in Figure S20). Combining the CSL model analysis and experimental values, we obtain noninteger values of (*m*, *n*) ≈ (7.12, 8.26) for *q*_1_ and (2.92, 3.38) for *q*_2_, indicating a clear deviation from a commensurate superlattice along the *a*-axis (Figure 5i). This weak interlayer registry indicates that it is less energetically favorable to force superlattice commensuration along the *a*-axis. For calculations, we assume that both the CoTe-epilayer and the TaCo_2_Te_2_-substrate lattices are rigid. Although this may not accurately represent our scenario, given that lattice instability exists above *T*_c_ and the heterostructure can relax via distortion. This explains why we did not observe independent Bragg reflections corresponding to the hexagonal Co_*x*_Te_*y*_ (0001) plane (Figure S21). While the Co_*x*_Te_*y*_ epilayer is directionally locked along the *b*-axis, translational symmetry breaking occurs along the *a*-axis to form a modulated epilayer structure, and Co_*x*_Te_*y*_ symmetry transforms from *C*_6_ to *C*_2_.

The resulting interlayer registry shows anisotropic interlayer coupling strength, with strong coupling forming rigid alignment along the *b*-axis and weak interlayer coupling along the lattice instability *a*-axis. Even with this weaker interlayer coupling, the lattice arrangement maintains precise values of *q*_1_ and *q*_2_ with minimal deviation across samples (Figure S22). Here, the dynamically varying periodic lattice potential along the lattice instability axis could be experienced by adatoms during growth. This anisotropic interlayer coupling results in directionally locked QvdW Co_*x*_Te_*y*_/TaCo_2_Te_2_ heteroepitaxy, which minimizes overall interfacial strain through lattice distortion along the lattice instability axis. Therefore, it prevents interfacial relaxation via rotational disorder at elevated temperatures. We elucidate this lattice instability-mediated interfacial stabilization by comparing the growth and interlayer registry of TaCo_2_Te_2_- and TaNi_2_Te_2_-based heterostructures. Because of the isostructural nature of TaNi_2_Te_2_, we expect a similar surface diffusion reaction in which Ta migrates to the surface and forms Ni–Te OSL, as discussed above in Figure 4. While we expect the interlayer registry to differ in the TaNi_2_Te_2_/Ni_*x*_Te_*y*_ heterostructure, we observe sample-to-sample variations of the superlattice structure. These variations manifest as diffuse, twinned superlattice peaks and new coherent peaks in SAED images at elevated temperatures (*T* > 700 K), unlike in TaCo_2_Te_2_ (Figure S23). Due to the absence of lattice instability and the presence of interlayer symmetry mismatch in the Ni_*x*_Te_*y*_/TaNi_2_Te_2_ heterostructure, the epilayer domains show a rotational degree of freedom to reach an energetically favorable configuration with increasing temperature.

### Interfacial Reconstruction Stabilizes Co_*x*_Te_*y*_/TaCo_2_Te_2_ Heterointerface

The effects of the lattice instability on the interlayer registry between the Co_*x*_Te_*y*_ and TaCo_2_Te_2_ are evident from the increased coherence of *q*_c_ peaks from diffuse SRO peaks above *T*_C_ in the SAED pattern, as shown in Figure 6a,b (additional samples are shown in Figure S24). This suggests the reentry of the distorted TaCo_2_Te_2_ structure. Analysis of the SAED pattern from the cross-section of bulk TaCo_2_Te_2_ along the zone axis [010], i.e., *ac* plane, shows no evidence of reentrant or superlattice peaks along a*, which confirms that the reentrant phase is not a TaCo_2_Te_2_ bulk phenomenon (Figure S25). Notably, these reentrant peaks maintain coherency even in the second heating cycle of the postcooled sample as well (Figure S24). Based on these observations, we conclude that there exists irreversible surface reconstruction back to RT distorted TaCo_2_Te_2_ above *T*_C,_ which assists the stability of Co_*x*_Te_*y*_/TaCo_2_Te_2_ heterointerface. The reentrant transition associated with Peierls distortion should result from the changes in the electron–phonon coupling at the Co_*x*_Te_*y*_/TaCo_2_Te_2_ heterointerface. Previous reports on the enhanced Peierls distortion in ultrathin bismuth films indicate that this phenomenon is affected by increased electron localization resulting from bond-confinement effects.^48^ We can expect increased localization of electron density of states at the QvdW interface, considering the electronic band structures, where CoTe is a semiconductor, and the undistorted TaCo_2_Te_2_ phase is a metal (Figure S26).^38,47^

**Figure 6:**
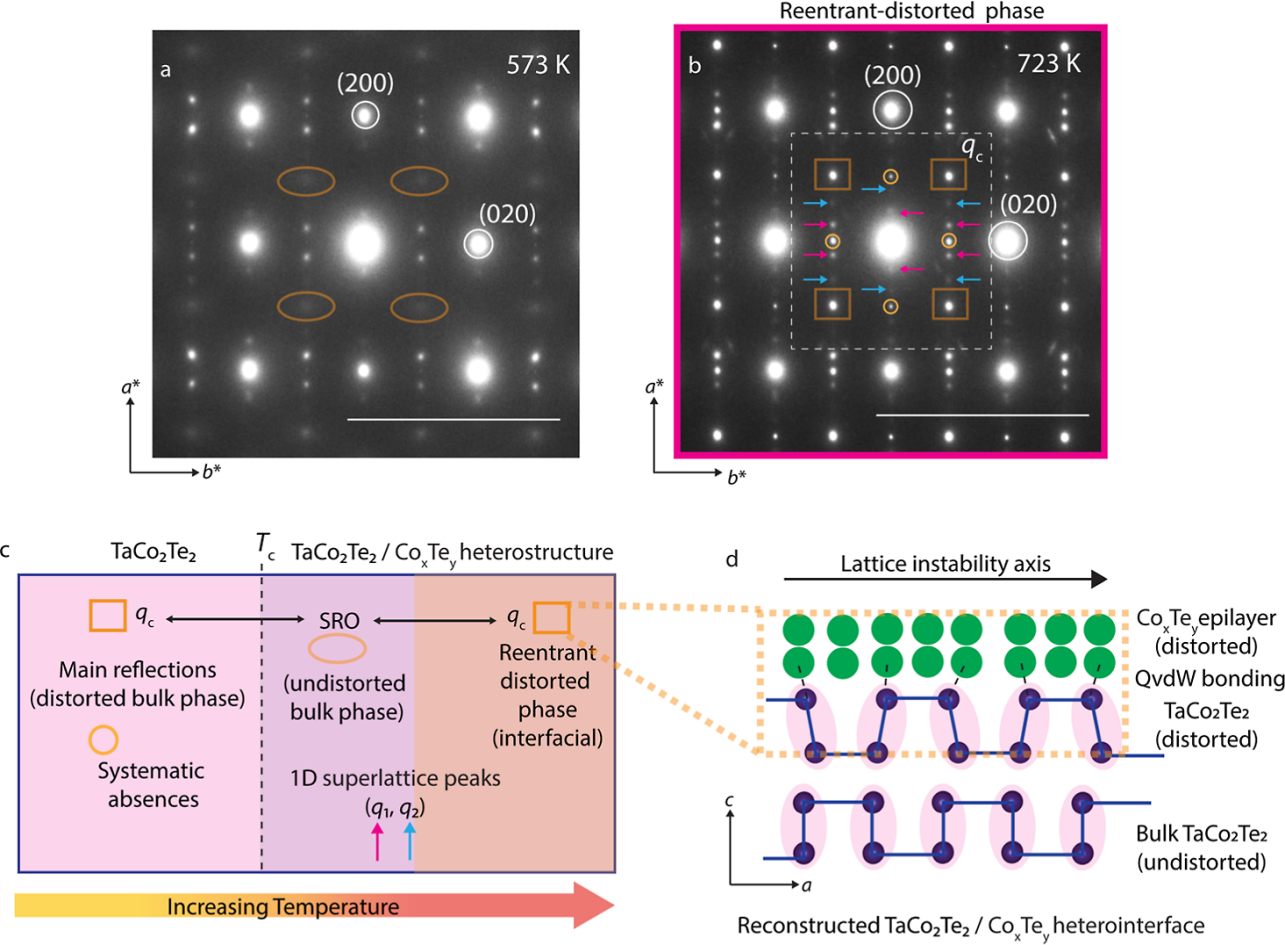
Reconstruction of TaCo_2_Te_2_/Co_*x*_Te_*y*_ heterointerface: (a,b) temperature evolution of TaCo_2_Te_2_ structure above *T*_C_ shown in in-plane SAED images. Orange ellipsoids and squares represent SRO and reentrant-distorted peaks, respectively. Dashed white box in panel b, highlighting the systematic absences (orange circles), 1D superlattice (pink and blue arrows), and reentrant-distorted peaks (orange squares). (c) Corresponding plot showing the structural phase evolution and emergence of superlattice peaks with increasing temperature. (d) Schematic showing the arrangement of the 1D modulated heterointerface with TaCo_2_Te_2_ surface reconstruction, while the rest of the bulk TaCo_2_Te_2_ remains undistorted above *T*_C_. Scale bar in panels a and b is 5 nm^–1^.

We summarize our observations in Figure 6c,d, which present schematics of the temperature evolution of diffraction peaks in in-plane SAED patterns of TaCo_2_Te_2_ nanoflakes (highlighting bulk and interfacial structural transitions). TaCo_2_Te_2_ stabilizes a Peierls-distorted bulk phase below *T*_c_, and we observe corresponding sharp peaks at *q*_c_. Above *T*_c_, the bulk phase transitions to a more stable undistorted phase and exhibits SRO peaks at *q*_c_ associated with anisotropic lattice fluctuations. In addition, we observe weak 1D superlattice peaks (*q*_1_, *q*_2_) along the lattice instability *a*-axis of TaCo_2_Te_2_, while the superlattice remains commensurate along the perpendicular *b*-axis. The intensities of *q*_1_ and *q*_2_ increase with temperature. The lattice instability facilitates lateral growth and directional locking of the modulated Co_*x*_Te_*y*_ epilayer on the TaCo_2_Te_2_ substrate. Finally, the reentrant peaks at *q*_c_ emerge without affecting the coherence of *q*_1_ and *q*_2_. The interlayer bond interactions at the QvdW Co_*x*_Te_*y*_/TaCo_2_Te_2_ interface along the lattice-locked *b*-axis should alter the TaCo_2_Te_2_ soft-phonon dynamics by suppressing lattice fluctuations. To maintain precise orientation lock-in between Co_*x*_Te_*y*_/TaCo_2_Te_2_, lattice fluctuations induced by increasing thermal energy can be further suppressed by surface distortion. Once sufficient epilayer coverage is achieved at high temperature, the reentrant distorted surface reconstruction peaks at *q*_c_ become coherent in SAED images. We finally observe peaks that deviate from the *a*-axis at high temperatures (723–753 K) in FIB-prepared samples. However, this does not compromise the coherence and positions of 1D superlattice peaks (Figure S27). In principle, the epilayer grown away from the QvdW interface should not experience the same lattice locking effects as the one closer to it. Therefore, rotation misalignment due to thermal relaxation becomes more likely as the epilayer thickness increases. The growth window and thickness for a lattice-locked epilayer using a structurally modulated substrate should be investigated in future studies. Our work encourages studies on predicting heterostructures with material combinations in which lattice instabilities can dictate growth and epilayer orientation.

## Conclusions

In summary, we show that the lattice instability of a structurally modulated TaCo_2_Te_2_ substrate can be leveraged to achieve robust epitaxial growth and precise alignment of the symmetry-mismatched bulk Co_*x*_Te_*y*_ epilayer. The dynamic lattice instabilities predicted by phonon calculations and confirmed through Raman and TEM measurements support anisotropic lattice fluctuations above *T*_C_ in TaCo_2_Te_2_. Atomic-resolution ADF-STEM images collected from postheat-treated cross-sectional samples reveal the growth progression of the crystalline Co_*x*_Te_*y*_ surface layer on TaCo_2_Te_2_. To stabilize the high-energy Co_*x*_Te_*y*_/TaCo_2_Te_2_ heterointerface, a coherent 1D incommensurate superlattice forms along the lattice instability axis, while strong lattice locking persists along the other orthorhombic axis. The emergence of the reentrant distorted phase far above *T*_C_ further lowers the overall interfacial energy by altering the soft-phonon dynamics of TaCo_2_Te_2_ at the heterointerface. Interfacial reconstruction arising from interactions between substrate lattice instability and QvdW bonding facilitates a structurally modulated interface, resulting in directionally locked heteroepitaxy even at elevated temperatures. Our work presents a strategy for robust multidimensional heteroepitaxy that leverages lattice instabilities to achieve precise interlayer registry by limiting rotational disorders and offers new design rules for deterministic assembly of symmetry-mismatched heterointerfaces.

## Methods

### Single Crystal Synthesis and Characterization

Bulk single crystals of TaCo_2_Te_2_ were synthesized using CVT. Synthesis of large single crystals of TaCo_2_Te_2_ was adopted from ref 38. Oriented single crystals with long axis along the *a*-direction prepared from elemental powder of Ta (Sigma-Aldrich 99.99%), Co (Sigma-Aldrich 99.9%) and Te (Sigma-Aldrich 99.999%), mixed in stoichiometric amount with 30 mg iodine chunks (Sigma-Aldrich 99.9%) and sealed in an evacuated quartz tube, which was heated to 1273 K for 10 days in a box furnace. Acquired mm-sized oriented TaCo_2_Te_2_ single crystals picked from the postreaction powder mixture using an optical microscope. Similarly, TaNi_2_Te_2_ single crystals were synthesized by replacing Co with Ni (Sigma-Aldrich 99.7%) powder. The sealed quartz tube was placed in a tube furnace for 10 days under a temperature gradient, with the powder mixture maintained at 875 °C at the hotter end and 775 °C at the colder end. After the reaction, TaNi_2_Te_2_ single crystals were collected at the hot and cold ends. The morphology and elemental composition of grown crystals were determined using Quanta 200 FEG ESEM equipped with energy dispersive X-ray (EDX) operating at 15 kV. Single-crystal X-ray diffraction (XRD) measurements were performed on single crystals <100 μm in size using a Bruker D8 VENTURE diffractometer with a PHOTON III CPAD detector and a graphite-monochromated Mo-*K*_α_ radiation source. All structure refinements were performed using the OLEX2 software package. Differential scanning calorimetry (DSC) experiments were conducted using a PerkinElmer DSC-8500.

### TaCo_2_Te_2_ Nanoflake Preparation

TaCo_2_Te_2_ single crystals were mechanically exfoliated using Scotch Magic tape (3 M). Prior to the transfer process, SiO_2_/Si substrates with a 285 nm oxide layer were thoroughly cleaned by ultrasonication with acetone, followed by the removal of organic residues using Ar/O_2_ (45/15 sccm) plasma cleaning (PE-50 PLASMA ETCH INC) for approximately 5 min. Following substrate preparation, a high density of laterally micron-sized vdW thin nanoflakes with varying thicknesses was transferred onto the cleaned SiO_2_/Si substrate. Atomic force microscopy (Bruker Dimension Icon AFM) was performed on thin exfoliated nanoflakes, and images were processed using NanoScope Analysis software. These flakes were picked up in ambient conditions from the SiO_2_/Si substrate using polymer stamps and a transfer stage equipped with micromanipulators. Glass-slide stamps were prepared using polycaprolactone (PCL, Sigma-Aldrich, *M*_n_:80000) solution (10% weight in chloroform), which was spin-coated onto the dome-shaped PDMS. Initially, a TEM Au grid (G2000HAG, Ted Pella INC.) was attached to a bare Si/SiO_2_ substrate using polycarbonyl (PC) solution. Multiple nanoflakes were then picked up and transferred onto the grid/Si substrate following the process detailed in ref 49. Subsequently, the entire grid/Si substrate assembly was immersed in a chloroform solution for 20 min to dissolve the post-transfer PC and PCL polymers. Finally, the TEM grid was carefully scooped from the chloroform solution and air-dried for 1 min.

### FIB Fabricated Thin Lamella

TEM thin lamellae were prepared using FIB cutting with a FEI Helios NanoLab 600 dual beam system (FIB/SEM). Oriented single crystals of TaCo_2_Te_2_ with their long axis along the *a*-axis were used as a guide to fabricate multiple lamellae for TEM measurements. These lamellae were cut both in-plane (*ab*-plane) and cross-sectionally (*ac*-planes) using FIB techniques. FIB lamella was attached to Mo grid (10GM02, Ted Pella PELCO) via in situ Pt deposition at shared edges. The cross-sectional STEM sample of the TaCo_2_Te_2_ nanoflake was prepared by transferring the nanoflake onto the top of the hBN nanoflake on a Si substrate. Next, a thin amorphous carbon layer was deposited on top of the TaCo_2_Te_2_/hBN assembly. Finally, a cross-section sample was prepared using FIB from amorphous carbon/TaCo_2_Te_2_/hBN/SiO_2_/Si assembly.

### TEM Microscopy

In situ heating TEM experiments were conducted using a Gatan double-tilt holder (Model 652) within the TEM column with a base pressure ≈10^–7^ Torr. Temperatures were set and monitored using a Gatan temperature controller (1905). After reaching the set temperature, the electron beam was blanked, and the sample was allowed to equilibrate for at least 20 min to minimize thermal drift before starting image collection. In situ cooling was achieved by setting the controller to 300 K (27 °C) and waiting overnight (∼15 h) before acquiring images. SAED and atomic resolution HAADF images were collected on a double Cs-corrected FEI Titan Cubed Themis 300 S/TEM. STEM images were processed using the Gatan Microscopy Suite software. The electron diffraction simulations were performed using the CrystalMaker and SingleCrystal software packages. Crystal structure schematics of TaCo_2_Te_2_ prepared using VESTA 3. ImageJ was used to generate intensity line scans of diffraction peaks in SAED images.

### Glovebox Raman Measurements

The air-stability of TaCo_2_Te_2_ exfoliated nanoflakes was determined using an argon glovebox Raman setup.^41^ Nanoflakes were first exfoliated onto a Si substrate in a glovebox. Raman spectra were collected immediately after exfoliation inside a glovebox and again following a 1 h exposure to ambient conditions. In situ Raman heating experiments were conducted on the same flakes in the same system. For both air-stability and temperature-dependent measurements, Raman spectra were acquired using a commercial WiTec Raman microscope. The system consists of a 100× objective lens and a fiber-coupled 532 nm laser set to 300 μW, with spectrometer integration times of 300 s. For the heating experiments, a custom heater stage consisting of a small aluminum block, a cartridge heater, and a thermocouple was used to heat the samples to 600 K. To ensure adequate thermal stability, the sample was held at each temperature for approximately 30 min before the next spectra were collected.

### STM Imaging

TaCo_2_Te_2_ samples were cleaved using Kapton tape at room temperature inside an ultrahigh vacuum (UHV) chamber with a base pressure of *p* ≈ 2 × 10^–10^ mbar. After cleaving, the samples were directly transferred into the STM head and cooled down to 4.2 K within 2 h. STM measurements were conducted using a home-built STM instrument under cryogenic (4 K ≤ T ≤ 24 K) and UHV (chamber pressure ≈2 × 10^–10^ mbar) conditions using a chemically etched tungsten tip which was prepared on a Cu(111) surface through field emission and controlled indentation, as well as calibrated against the Cu(111) Shockley surface state before each set of measurements. Topographies were recorded using constant current (*I*) mode. STM topography of TaCo_2_Te_2_ surface was obtained by using bias voltage (*V*_b_) = −5 mV, *I* = 4 nA.

### First-Principles Electronic and Phonon Calculations

Density functional theory (DFT) calculations were performed to calculate the electronic band structures of TaCo_2_Te_2_ using the Vienna Ab initio Simulation Package (VASP) *v*5.4.4 using the PBE functional. PAW potentials were chosen based on recommended potentials. We used *a* plane wave energy cutoff of 600 eV. We used Γ-centered 15 × 15 × 5 and 30 × 15 × 5 *k*-meshes for the undistorted and RT TaCo_2_Te_2_ structures, respectively. The atomic positions of the undistorted TaCo_2_Te_2_ structure were allowed to relax with an energy convergence criterion of 10^–7^ eV. A static calculation was performed on each structure using an energy convergence criterion of 10^–7^ eV. Gaussian smoothing was applied to the density of states (DOS) calculation. DFT calculations were performed to calculate phonon band structures of TaCo_2_Te_2_ compounds using simulations using the QuantumESPRESSO package and the PBEsol functionals found in the QuantumESPRESSO pseudopotential database.^50,51^ For TaCo_2_Te_2_, an energy cutoff of 65 Ry (Ry), together with a 12 × 6 × 3 *k*-mesh for the self-consistent field (SCF) calculations. An energy threshold of 10^–10^ Ry was used for the SCF. Density functional perturbation theory calculations were performed using a 2 × 1 × 1 *q*-mesh and an energy threshold of 10^–20^ Ry to determine phonon spectra.

## Supplementary Material











## Notes

CoTe.cif (CIF)
The Supporting Information is available and includes additional figures, tables, and supplementary notes on the detailed structural characterization of the TaCo_2_Te_2_ single crystal, TEM sample fabrication methods, additional atomic-resolution STEM images, in situ TEM studies and diffraction analyses on multiple samples, coincidence site lattice analysis, and DFT calculations (PDF)
TaCo2Te2_Peierls_distorted.cif (CIF)
TaCo2Te2_vesta_undistorted_hightemp.cif (CIF)
TaNi2Te2.cif (CIF)

## References

[1] Das S., Sebastian A., Pop E., McClellan C. J., Franklin A. D., Grasser T., Knobloch T., Illarionov Y., Penumatcha A. V., Appenzeller J., Chen Z., Zhu W., Asselberghs I., Li L.-J., Avci U. E., Bhat N., Anthopoulos T. D., Singh R. (2021). Transistors based on two-dimensional
materials for future integrated circuits. Nat.
Electron..

[2] Liu W., Yu Y., Peng M., Zheng Z., Jian P., Wang Y., Zou Y., Zhao Y., Wang F., Wu F., Chen C., Dai J., Wang P., Hu W. (2023). Integrating 2D layered materials
with 3D bulk materials as van der Waals heterostructures for photodetections:
Current status and perspectives. InfoMat.

[3] Xu X., Guo T., Kim H., Hota M. K., Alsaadi R. S., Lanza M., Zhang X., Alshareef H. N. (2022). Growth of 2D Materials at the Wafer
Scale. Adv. Mater..

[4] Choudhury T. H., Zhang X., Al Balushi Z. Y., Chubarov M., Redwing J. M. (2020). Epitaxial
Growth of Two-Dimensional Layered Transition Metal Dichalcogenides. Annu. Rev. Mater. Res..

[5] Zhang Z., Yang X., Liu K., Wang R. (2022). Epitaxy of 2D Materials
toward Single Crystals. Adv. Sci..

[6] Liang D., Wei T., Wang J., Li J. (2020). Quasi van der Waals epitaxy nitride
materials and devices on two dimension materials. Nano Energy.

[7] Reidy K., Varnavides G., Thomsen J. D., Kumar A., Pham T., Blackburn A. M., Anikeeva P., Narang P., LeBeau J. M., Ross F. M. (2021). Direct
imaging and electronic structure modulation
of moiré superlattices at the 2D/3D interface. Nat. Commun..

[8] Shi Y., Zhou W., Lu A.-Y., Fang W., Lee Y.-H., Hsu A. L., Kim S. M., Kim K. K., Yang H. Y., Li L.-J., Idrobo J.-C., Kong J. (2012). van der Waals Epitaxy
of MoS2 Layers Using Graphene As Growth Templates. Nano Lett..

[9] Miwa J. A., Dendzik M., Grønborg S. S., Bianchi M., Lauritsen J. V., Hofmann P., Ulstrup S. (2015). Van der Waals
Epitaxy of Two-Dimensional
MoS2-Graphene Heterostructures in Ultrahigh Vacuum. ACS Nano.

[10] Kim S., Oh S., Kwak S. J., Noh G., Choi M., Lee J., Kim Y., Kim M.-g., Kim T. S., Jo M.-k., Lee W. B., Yoo J., Hong Y. J., Song S., Kwak J. Y., Kim Y., Jeong H. Y., Kang K. (2025). Sequential multidimensional heteroepitaxy
of chalcogen-sharing 3D ZnSe and 2D MoSe2 with quasi van der Waals
interface engineering. Sci. Adv..

[11] Grønborg S. S., Ulstrup S., Bianchi M., Dendzik M., Sanders C. E., Lauritsen J. V., Hofmann P., Miwa J. A. (2015). Synthesis
of Epitaxial
Single-Layer MoS2 on Au(111). Langmuir.

[12] Reidy K., Thomsen J. D., Lee H. Y., Zarubin V., Yu Y., Wang B., Pham T., Periwal P., Ross F. M. (2022). Mechanisms
of Quasi van der Waals Epitaxy of Three-Dimensional Metallic Nanoislands
on Suspended Two-Dimensional Materials. Nano
Lett..

[13] Koda D. S., Bechstedt F., Marques M., Teles L. K. (2016). Coincidence Lattices
of 2D Crystals: Heterostructure Predictions and Applications. J. Phys. Chem. C.

[14] Grundmann M., Böntgen T., Lorenz M. (2010). Occurrence of Rotation Domains in
Heteroepitaxy. Phys. Rev. Lett..

[15] Dong J., Liu Y., Ding F. (2022). Mechanisms
of the epitaxial growth of two-dimensional
polycrystals. npj Comput. Mater..

[16] Liu L., Siegel D. A., Chen W., Liu P., Guo J., Duscher G., Zhao C., Wang H., Wang W., Bai X., McCarty K. F., Zhang Z., Gu G. (2014). Unusual role of epilayer-substrate
interactions in determining orientational relations in van der Waals
epitaxy. Proc. Natl. Acad. Sci. U.S.A.

[17] Hogan C., Holtgrewe K., Ronci F., Colonna S., Sanna S., Moras P., Sheverdyaeva P. M., Mahatha S., Papagno M., Aliev Z. S., Babanly M., Chulkov E. V., Carbone C., Flammini R. (2019). Temperature Driven
Phase Transition at the Antimonene/Bi2Se3
van der Waals Heterostructure. ACS Nano.

[18] Liu F., Wang T., Zhang Z., Shen T., Rong X., Sheng B., Yang L., Li D., Wei J., Sheng S., Li X., Chen Z., Tao R., Yuan Y., Yang X., Xu F., Zhang J., Liu K., Li X.-Z., Shen B., Wang X. (2022). Lattice Polarity Manipulation
of Quasi-vdW Epitaxial GaN Films on Graphene Through Interface Atomic
Configuration. Adv. Mater..

[19] Cohen A., Mohapatra P. K., Hettler S., Patsha A., Narayanachari K. V. L. V., Shekhter P., Cavin J., Rondinelli J. M., Bedzyk M., Dieguez O., Arenal R., Ismach A. (2023). Tungsten Oxide
Mediated Quasi-van der Waals Epitaxy of WS2 on Sapphire. ACS Nano.

[20] Zallo E., Cecchi S., Boschker J. E., Mio A. M., Arciprete F., Privitera S., Calarco R. (2017). Modulation of van der Waals and classical
epitaxy induced by strain at the Si step edges in GeSbTe alloys. Sci. Rep..

[21] Jang M., Kim M., Lee S., Kwon M., Kang H., Lee K., Park J., Hoang A. T., Ahn J.-H., Lee Y., Kim K. (2024). Controlled
epitaxy and patterned growth of one-dimensional crystals
via surface treatment of two-dimensional templates. npj 2D Mater. Appl..

[22] Lin Z., Yin A., Mao J., Xia Y., Kempf N., He Q., Wang Y., Chen C.-Y., Zhang Y., Ozolins V., Ren Z., Huang Y., Duan X. (2016). Scalable solution-phase epitaxial
growth of symmetry-mismatched heterostructures on two-dimensional
crystal soft template. Sci. Adv..

[23] Janssen T., Janner A. (2014). Aperiodic crystals and superspace concepts. Acta Crystallogr. Sect. B:Struct. Sci..

[24] de
Wolff P. M., Janssen T., Janner A. (1981). The superspace groups
for incommensurate crystal structures with a one-dimensional modulation. Acta Crystallogr., Sect. A:.

[25] Pinheiro C. B., Abakumov A. M. (2015). Superspace crystallography: a key
to the chemistry
and properties. IUCrJ.

[26] Gao S., Flicker F., Sankar R., Zhao H., Ren Z., Rachmilowitz B., Balachandar S., Chou F., Burch K. S., Wang Z., van Wezel J., Zeljkovic I. (2018). Atomic-scale
strain manipulation of a charge density wave. Proc. Natl. Acad. Sci. U.S.A.

[27] Devarakonda A., Chen A., Fang S., Graf D., Kriener M., Akey A. J., Bell D. C., Suzuki T., Checkelsky J. G. (2024). Evidence
of striped electronic phases in a structurally modulated superlattice. Nature.

[28] Chatterjee U., Zhao J., Iavarone M., Di Capua R., Castellan J. P., Karapetrov G., Malliakas C. D., Kanatzidis M. G., Claus H., Ruff J. P. C., Weber F., van Wezel J., Campuzano J. C., Osborn R., Randeria M., Trivedi N., Norman M. R., Rosenkranz S. (2015). Emergence of coherence in the charge-density
wave state of 2H-NbSe2. Nat. Commun..

[29] Rossnagel K. (2011). On the origin
of charge-density waves in select layered transition-metal dichalcogenides. J. Phys.: Condens. Matter.

[30] Langmann J., Haas C., Wenger E., Schaniel D., Scherer W., Eickerling G. (2020). Evidence for
a soft phonon mode driven Peierls-type
distortion in Sc3CoC4. Phys. Rev. B.

[31] Jia R., Xin Y., Potter M., Jiang J., Wang Z., Ma H., Zhang Z., Liang Z., Zhang L., Lu Z., Yang R., Pendse S., Hu Y., Peng K., Meng Y., Bao W., Liu J., Wang G.-C., Lu T.-M., Shi Y., Gao H., Shi J. (2025). Long-distance
remote epitaxy. Nature.

[32] Pandya S., Damodaran A. R., Xu R., Hsu S.-L., Agar J. C., Martin L. W. (2016). Strain-induced growth instability
and nanoscale surface
patterning in perovskite thin films. Sci. Rep..

[33] Galvis J. A., Fang A., Jiménez-Guerrero D., Rojas-Castillo J., Casas J., Herrera O., Garcia-Castro A. C., Bousquet E., Fisher I. R., Kapitulnik A., Giraldo-Gallo P. (2023). Nanoscale phase-slip domain walls in the charge density
wave state of the Weyl semimetal candidate NbTe4. Phys. Rev. B.

[34] Siddique S., Hart J. L., Niedzielski D., Singha R., Han M.-G., Funni S. D., Colletta M., Kiani M. T., Schnitzer N., Williams N. L., Kourkoutis L. F., Zhu Y., Schoop L. M., Arias T. A., Cha J. J. (2024). Realignment and
suppression of charge
density waves in the rare-earth tritellurides RTe3 (R=La, Gd, Er). Phys. Rev. B.

[35] Sung S. H., Agarwal N., El Baggari I., Kezer P., Goh Y. M., Schnitzer N., Shen J. M., Chiang T., Liu Y., Lu W., Sun Y., Kourkoutis L. F., Heron J. T., Sun K., Hovden R. (2024). Endotaxial stabilization of 2D charge density waves
with long-range order. Nat. Commun..

[36] Cao S., Xu C., Fukui H., Manjo T., Dong Y., Shi M., Liu Y., Cao C., Song Y. (2023). Competing charge-density
wave instabilities
in the kagome metal ScV6Sn6. Nat. Commun..

[37] Tremel W. (1992). TaNi2Te2,
A Novel Layered Telluride, and TaCo2Te2, a Structural Variant with
Peierls Distortion. Angew. Chem., Int. Ed. Engl..

[38] Singha R., Yuan F., Cheng G., Salters T. H., Oey Y. M., Villalpando G. V., Jovanovic M., Yao N., Schoop L. M. (2022). TaCo2Te2:
An Air-Stable, High Mobility Van der Waals Material with Probable
Magnetic Order. Adv. Funct. Mater..

[39] Husremović S., Goodge B. H., Erodici M. P., Inzani K., Mier A., Ribet S. M., Bustillo K. C., Taniguchi T., Watanabe K., Ophus C., Griffin S. M., Bediako D. K. (2023). Encoding
multistate charge order and chirality in endotaxial heterostructures. Nat. Commun..

[40] Pallikara I., Kayastha P., Skelton J. M., Whalley L. D. (2022). The physical significance
of imaginary phonon modes in crystals. Electron.
Struct..

[41] Gray M. J., Kumar N., O’Connor R., Hoek M., Sheridan E., Doyle M. C., Romanelli M. L., Osterhoudt G. B., Wang Y., Plisson V., Lei S., Zhong R., Rachmilowitz B., Zhao H., Kitadai H., Shepard S., Schoop L. M., Gu G. D., Zeljkovic I., Ling X., Burch K. S. (2020). A cleanroom in a glovebox. Rev. Sci. Instrum..

[42] Tian Y., Jia S., Cava R. J., Zhong R., Schneeloch J., Gu G., Burch K. S. (2017). Understanding
the evolution of anomalous anharmonicity
in Bi2Te3–xSex. Phys. Rev. B.

[43] Bertrang K., Hinke T., Kaiser S., Knechtges M., Loi F., Lacovig P., Jahangirzadeh Varjovi M., Esch F., Baraldi A., Tosoni S., Kartouzian A., Heiz U. (2025). The Interaction of Sub-Monolayer Ta Adatoms and Clusters with Oxygen
at the Pt(111) Interface. J. Phys. Chem. C.

[44] Cui W., Lin W., Lu W., Liu C., Gao Z., Ma H., Zhao W., Van Tendeloo G., Zhao W., Zhang Q., Sang X. (2023). Direct observation of cation diffusion driven surface reconstruction
at van der Waals gaps. Nat. Commun..

[45] Levi A. C., Kotrla M. (1997). Theory and simulation of crystal
growth. J. Phys.: Condens. Matter.

[46] Hu L., Liu D., Zheng F., Yang X., Yao Y., Shen B., Huang B. (2024). Hybrid van der Waals Epitaxy. Phys. Rev. Lett..

[47] Liu Y., Gong Q., Yin Y., Yi M., Liu Y. (2024). Emerging 2D
Cobalt Telluride (CoxTey): from Theory to Applications. Adv. Funct. Mater..

[48] Hoff F., Kerres P., Veslin T., Jalil A. R., Schmidt T., Ritarossi S., Köttgen J., Bothe L., Frank J., Schön C.-F., Xu Y., Kim D., Mertens J., Mayer J., Mazzarello R., Wuttig M. (2025). Bond Confinement-Dependent
Peierls Distortion in Epitaxially Grown Bismuth Films. Adv. Mater..

[49] Son S., Shin Y. J., Zhang K., Shin J., Lee S., Idzuchi H., Coak M. J., Kim H., Kim J., Kim J. H., Kim M., Kim D., Kim P., Park J.-G. (2020). Strongly adhesive dry transfer technique for van der
Waals heterostructure. 2D Mater..

[50] Giannozzi P., Andreussi O., Brumme T., Bunau O., Buongiorno
Nardelli M., Calandra M., Car R., Cavazzoni C., Ceresoli D., Cococcioni M., Colonna N., Carnimeo I., Dal Corso A., de Gironcoli S., Delugas P., DiStasio R. A., Ferretti A., Floris A., Fratesi G., Fugallo G., Gebauer R. (2017). Advanced capabilities for materials modelling with
Quantum ESPRESSO. J. Phys.: Condens. Matter.

[51] Giannozzi P., Baroni S., Bonini N., Calandra M., Car R., Cavazzoni C., Ceresoli D., Chiarotti G. L., Cococcioni M., Dabo I., Dal Corso A., de Gironcoli S., Fabris S., Fratesi G., Gebauer R., Gerstmann U., Gougoussis C., Kokalj A., Lazzeri M., Martin-Samos L., Marzari N. (2009). QUANTUM ESPRESSO: a
modular and open-source software project for quantum simulations of
materials. J. Phys.: Condens. Matter.

