# Supplementary material for: Directionally Locked Heteroepitaxy with a Structurally Modulated van der Waals Material
Source: ACS Nano. 2026 Jun 23;20(26):18780–91. doi: 10.1021/acsnano.6c04146 (PMC13348181; doi:10.1021/acsnano.6c04146)
Supplement: Supplementary file 2 [file nn6c04146_si_002.pdf]

Supplementary Information for

Directionally Locked Heteroepitaxy with a Structurally Modulated van der Waals Material

Nitish Mathur[1], Guangming Cheng[2], Francesc Ballester[3,4], Gabrielle Carrel[1], Vincent M. Plisson[5], Fang Yuan[1], Jiangchang Zheng[6], Caiyun Chen[6], Scott B. Lee[1], Ratnadwip Singha[1,7], Sudipta Chatterjee[1], Kenji Watanabe[8], Takashi Taniguchi[9], Kenneth S. Burch[5], Berthold Jäck[6], Ion Errea[3,4,10], Maia G. Vergniory[3,11], Nan Yao[2], Sanfeng Wu[12], and Leslie M. Schoop[1*]

1. Department of Chemistry, Princeton University, Princeton, NJ 08544, USA
2. Princeton Materials Institute, Princeton, NJ 08544, USA
3. Donostia International Physics Center, San Sebastián 20018, Spain
4. Department of Applied Physics, University of the Basque Country (UPV/EHU), San Sebastián 20018, Spain
5. Department of Physics, Boston College, Chestnut Hill, MA 02467, USA
6. Department of Physics, The Hong Kong University of Science and Technology, Clear Water Bay, Kowloon 999077, Hong Kong
7. Department of Physics, Indian Institute of Technology Guwahati, Assam 781039, India
8. Research Center for Electronic and Optical Materials, National Institute for Materials Science, 1-1 Namiki, Tsukuba 305-0044, Japan
9. Research Center for Materials Nanoarchitectonics, National Institute for Materials Science, 1-1 Namiki, Tsukuba 305-0044, Japan
10. Centro de Física de Materiales (CSIC-UPV/EHU), San Sebastián 20018, Spain
11. Département de Physique et Institut Quantique, Université de Sherbrooke, Sherbrooke, Québec J1K 2R1, Canada
12. Department of Physics, Princeton University, Princeton, New Jersey 08544, USA

Corresponding author* (email): lschoop@princeton.edu

Table S1. Crystallographic information for $TaCo_2Te_2$ obtained from single-crystal X-ray diffraction.

Refined Composition	$TaCo_2Te_2$
Crystal radius (mm^{-1})	0.045
Radiation source, λ (Å)	Mo $K_{\alpha1}$, 0.70103
Data Collection Temperature (K)	293
Space Group	*Pnma* (62)
a (Å)	17.7851(7)
b (Å)	6.5877(3)
c (Å)	6.5665(3)
Cell Volume ($Å^3$)	769.35(6)
Absorption Coefficient (mm^{-1})	51.514
θ_{min}, θ_{max} (deg)	2.29, 45.33
Refinement Method	F^2
R_{int} (I>3σ, all)	6.80, 7.14
Number of Parameters	53
Unique Reflections (I>3σ, all)	2732, 3419
R (I>3σ), R_w(I>3σ)	0.0432, 0.1200
R(all), R_w(all)	0.0545, 0.1219
S (I>3σ), S(all)	2.7030, 2.4526
$\Delta\rho_{max}$, $\Delta\rho_{min}$ (e $Å^{-3}$)	4.83, -2.67

Table S2. Refined atomic coordinates for $TaCo_2Te_2$.

Site	Wyckoff Position	x	y	z	U_{ani}
Ta1	4c	0.778211(18)	¼	0.34968(6)	0.00477(7)
Ta2	4c	0.735085(17)	¼	0.84904(5)	0.00419(7)
Te1	4c	0.58647(3)	¼	0.35040(9)	0.00549(11)
Te2	4c	0.89296(3)	¼	0.84652(9)	0.00588(11)
Te3	8d	0.90636(2)	0.50299(5)	0.34930(6)	0.00557(8)
Co1	8d	0.68106(4)	0.46577(12)	0.54388(14)	0.00487(15)
Co2	8d	0.68103(4)	0.03539(13)	0.15471(13)	0.00522(16)

Table S3. Refined anisotropic displacement parameters for $TaCo_2Te_2$.

Site	U_{11}	U_{22}	U_{33}	U_{12}	U_{13}	U_{23}
Ta1	0.00342(11)	0.00508(12)	0.00581(13)	0	0.00002(9)	0
Ta2	0.00303(11)	0.00489(12)	0.00465(12)	0	0.00003(9)	0
Te1	0.00389(17)	0.00604(19)	0.00655(19)	0	-0.00023(14)	0
Te2	0.00386(17)	0.00596(19)	0.0078(2)	0	0.00005(15)	0
Te3	0.00313(12)	0.00781(15)	0.00576(14)	0.00004(9)	-0.00011(10)	-0.00016(11)
Co1	0.0036(2)	0.0064(3)	0.0046(3)	0.0001(2)	0.0001(2)	0.0001(2)
Co2	0.0038(3)	0.0066(3)	0.0053(3)	0.0003(2)	0.0004(2)	-0.0002(2)

Table S4. Select interatomic distances for $TaCo_2Te_2$

Site	Neighbor	Multiplicity	Distance
Ta1	Te3	2	2.8234(5)
Ta1	Co1	2	2.5753(9)
Ta1	Co1	2	2.8395(9)
Ta1	Co2	2	2.5740(9)
Ta1	Co2	2	2.8411(9)
Ta2	Te2	1	2.8079(6)
Ta2	Co1	2	2.6380(9)
Ta2	Co1	2	2.7142(9)
Ta2	Co2	2	2.6366(9)
Ta2	Co2	2	2.7183(9)
Te1	Co1	2	2.5426(9)
Te1	Co2	2	2.5452(10)
Te2	Co1	2	2.6303(9)
Te2	Co2	2	2.6178(9)
Te3	Co1	1	2.5459(10)
Te3	Co2	1	2.5461(9)

Supplementary Note 1. $TaCo_2Te_2$ single-crystal data are refined with the standard CIF file with space group convention *Pnma* (62) (a = 1.77851 nm, b = 0.65877 nm, c = 0.65665 nm). The convention is converted to *Pmcn* (62) ($a \approx 0.660$ nm, $b \approx 0.658$ nm, $c \approx 1.78$ nm) in the main text.

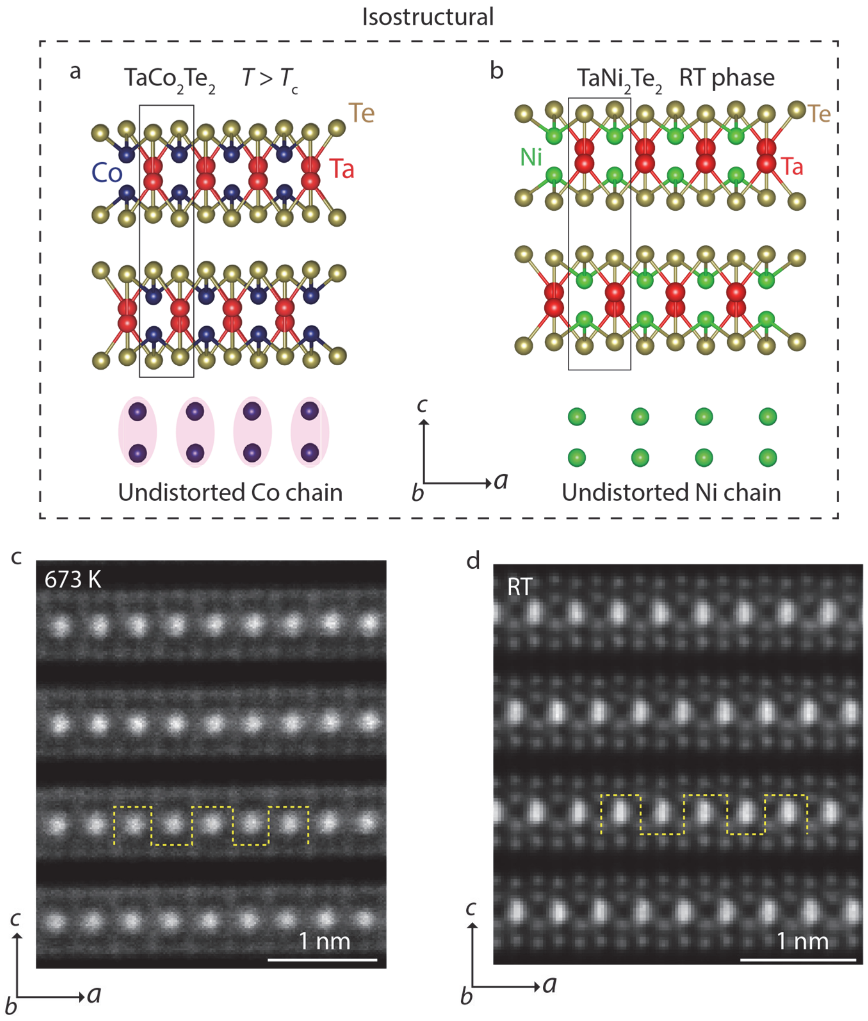

Figure S1. Isostructural undistorted $TaCo_2Te_2$ and $TaNi_2Te_2$**.** Schematic depicting the isostructural (**a**) $TaCo_2Te_2$ above T_C and (**b**) $TaNi_2Te_2$ at room temperature (RT) viewed along the *b*-axis. Corresponding atomic resolution cross-sectional STEM images of (**c**) $TaCo_2Te_2$ at 673 (> T_C) and (**d**) $TaNi_2Te_2$ at RT. Dotted yellow lines highlight the undistorted arrangement of the Co atomic chain along the *a*-axis.

Supplementary Note 2. The crystallographic information file for the undistorted $TaCo_2Te_2$ structure ($a \approx 0.330$ nm , $b \approx 0.658$ nm, $c \approx 1.78$ nm) above T_C was derived from the standard unit cell of the isostructural RT orthorhombic $TaNi_2Te_2$ ($a = 0.3566$ nm, $b = 0.6488$ nm, $c = 1.7014$ nm, space group *Pmnb*, 62). To obtain the undistorted structure, the lattice parameters were first adjusted to match the distorted standard unit cell of $TaCo_2Te_2$ ($a \approx 0.660$ nm, $b \approx 0.658$ nm, $c \approx 1.78$ nm) along the *b*- and *c*-axes, followed by halving the unit cell along the *a*-axis. Both structures share the same space group (No. 62).

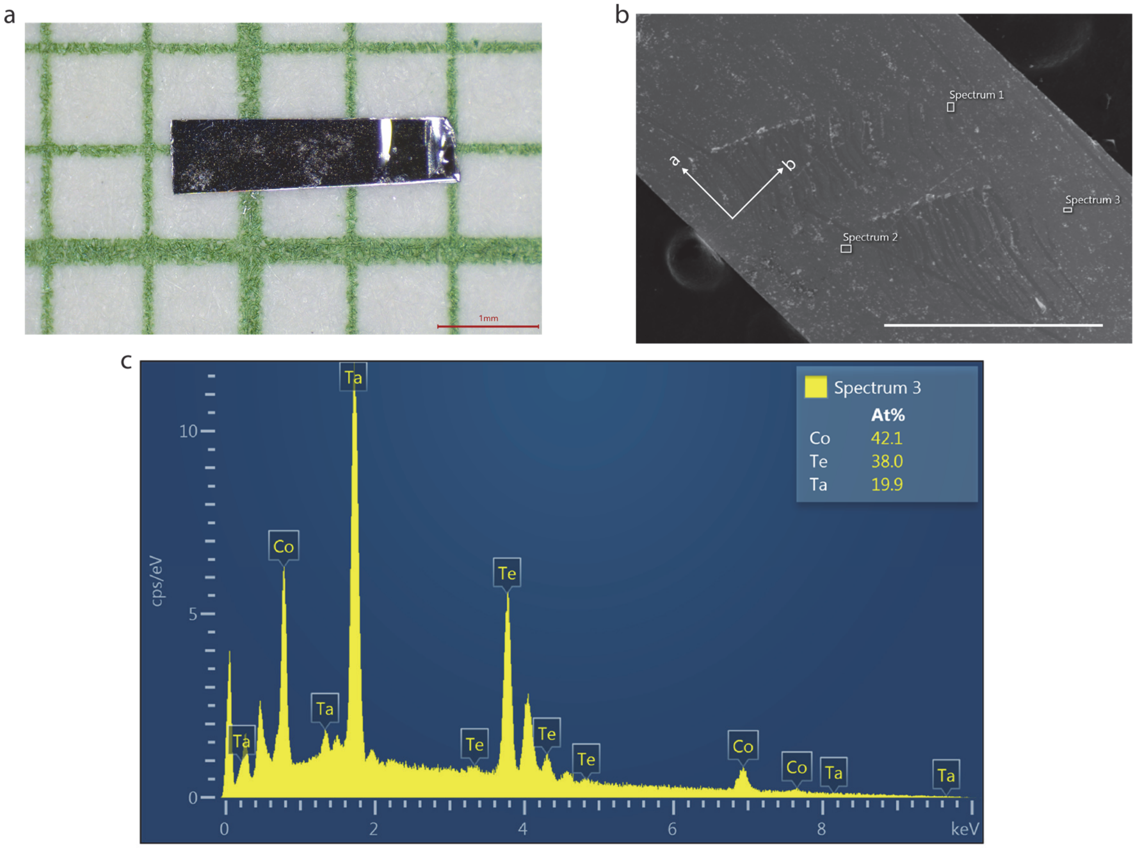

Figure S2. Oriented $TaCo_2Te_2$ crystals synthesized *via* chemical vapor transport. **(a)** Optical microscope image of a millimeter-sized $TaCo_2Te_2$ crystal. (**b**) SEM image of the crystal used for FIB-cut TEM samples. (**c**) Representative SEM-EDS spectrum of the analysis area demonstrating the presence of Ta, Co, and Te elements. Scale bar in panel b is 0.5 mm.

Table S5. Compositional analysis of a $TaCo_2Te_2$ single crystal. The atomic % of each element was determined from 3 regions of the $TaCo_2Te_2$ single crystal, shown in Figure S2b.

Spectrum	Atomic % Ta	Atomic % Co	Atomic % Te	Ratio (Ta: Co: Te)
1	19.4	43.2	37.5	1: 2.21: 1.93
2	19.4	42.4	38.0	1: 2.18: 1.95
3	19.9	42.1	38.0	1: 2.11: 1.90

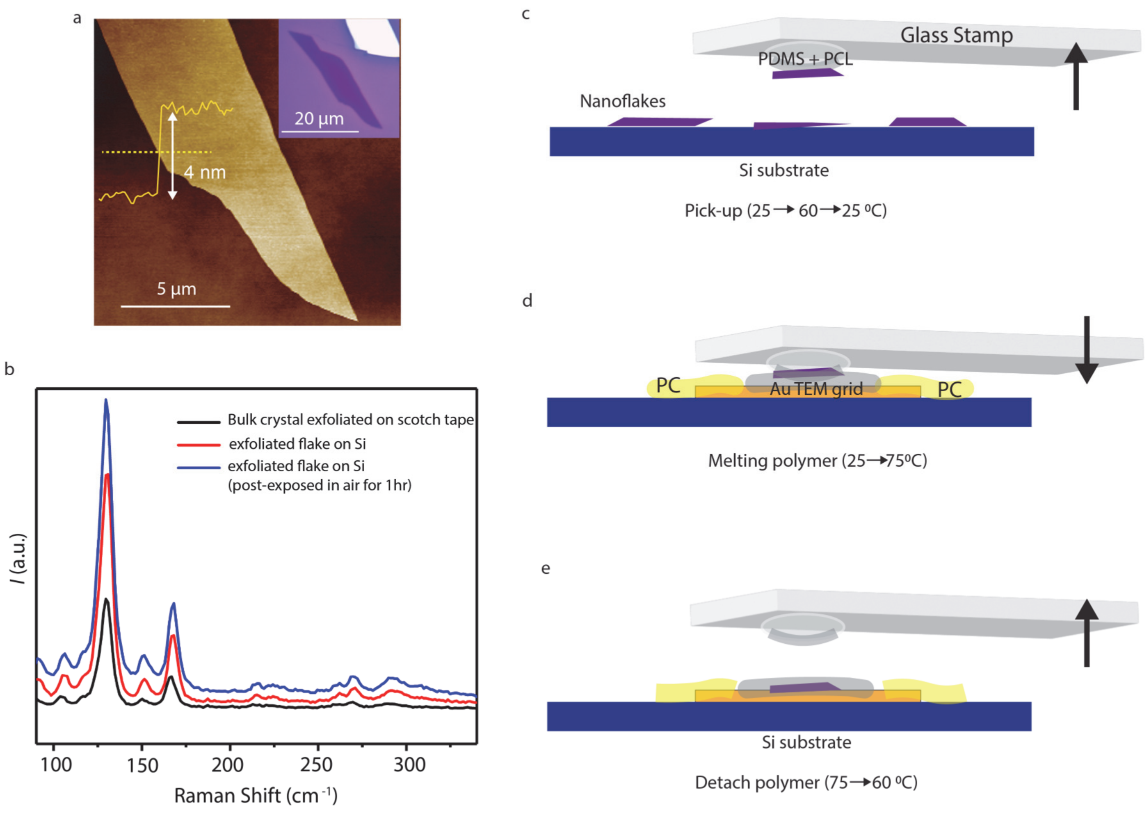

Figure S3. Exfoliation, air-stability, and polymer-transfer method of $TaCo_2Te_2$ exfoliated nanoflakes. (**a**) AFM image of a $TaCo_2Te_2$ thin flake (thickness ≈ 4 nm). Inset showing the optical microscope image of the nanoflake on a Si/SiO_2 (285 nm) substrate. (**b**) Glovebox Raman spectroscopy demonstrates the air stability of exfoliated nanoflakes post-exfoliation. Raman spectra show no new peaks even after 1 hour of air exposure following glovebox exfoliation. (**c-e**) Schematics illustrating the complete pick-up and transfer process of $TaCo_2Te_2$ nanoflakes onto an Au TEM grid using PCL/PDMS glass stamps. PC was used to secure the thin TEM grid to the Si substrate due to its higher glass transition temperature than PCL. Schematics indicate the temperature ranges applied at each step of the process.

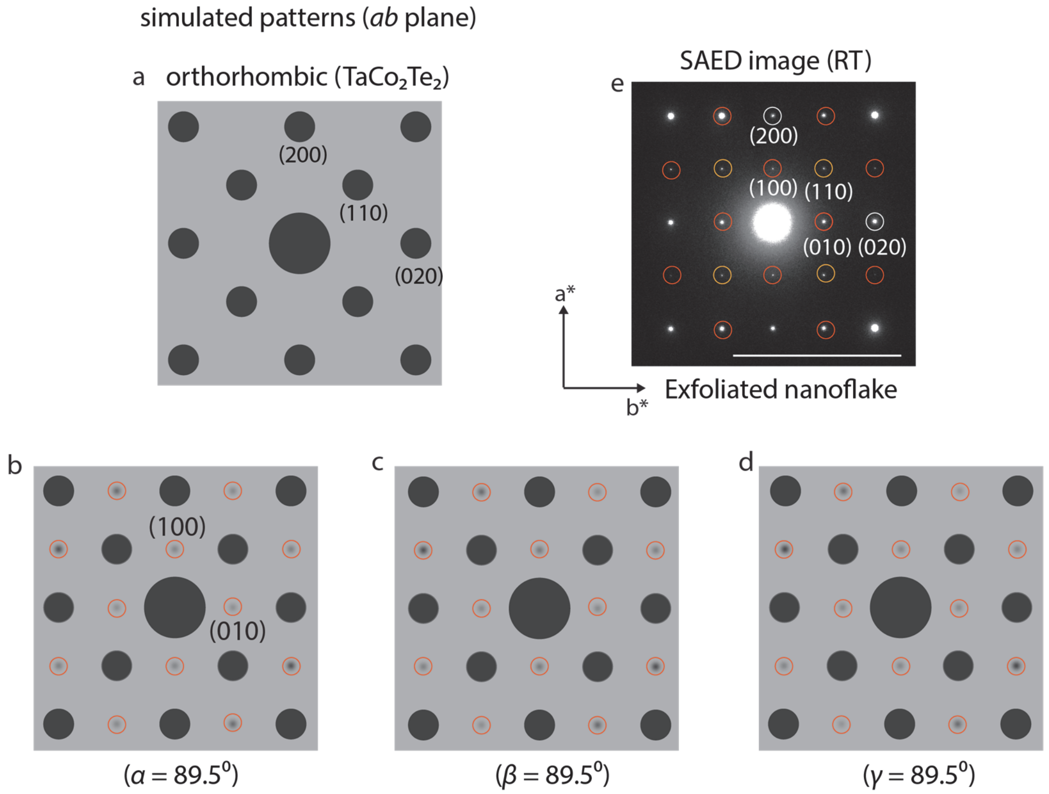

Figure S4. Appearance of forbidden reflections in TEM diffraction patterns of $TaCo_2Te_2$ nanoflakes. (**a**) The simulated *ab*-plane TEM diffraction of orthorhombic distorted $TaCo_2Te_2$. (**b-d**) Simulated *ab*-plane TEM diffractions revealing weak forbidden reflections resulting from a minor monoclinic distortion between any lattice vectors. (**e**) SAED image of a $TaCo_2Te_2$ nanoflake with Bragg-main, Peierls-distorted, and forbidden reflections marked with white, orange, and red circles, respectively. Scale bar in panel e is 5 nm^{-1}.

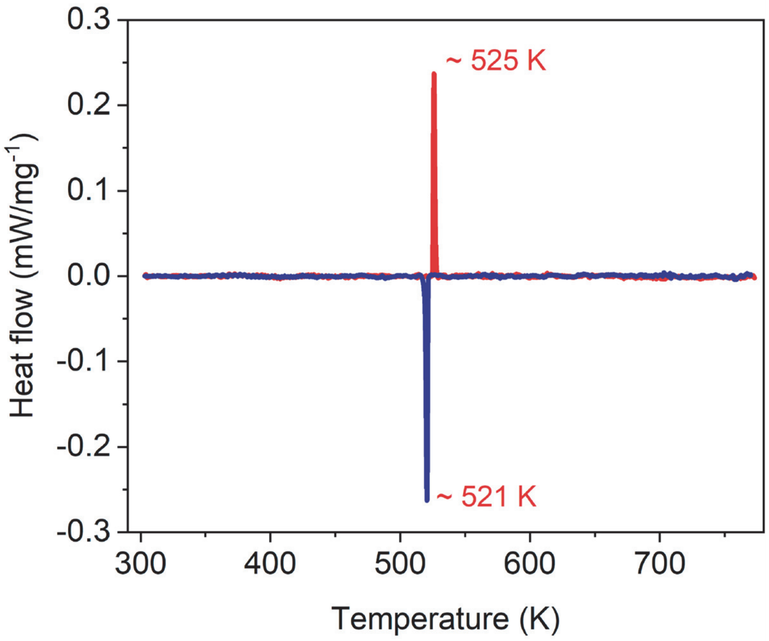

Figure S5. Differential scanning calorimetry (DSC) scans of a $TaCo_2Te_2$ single crystal. Scans are background-subtracted for clarity. Red and blue lines correspond to heating and cooling scans, respectively. T_c is calculated as the average temperature at which the peaks appeared in the heating and cooling scans, i.e., 523 K.

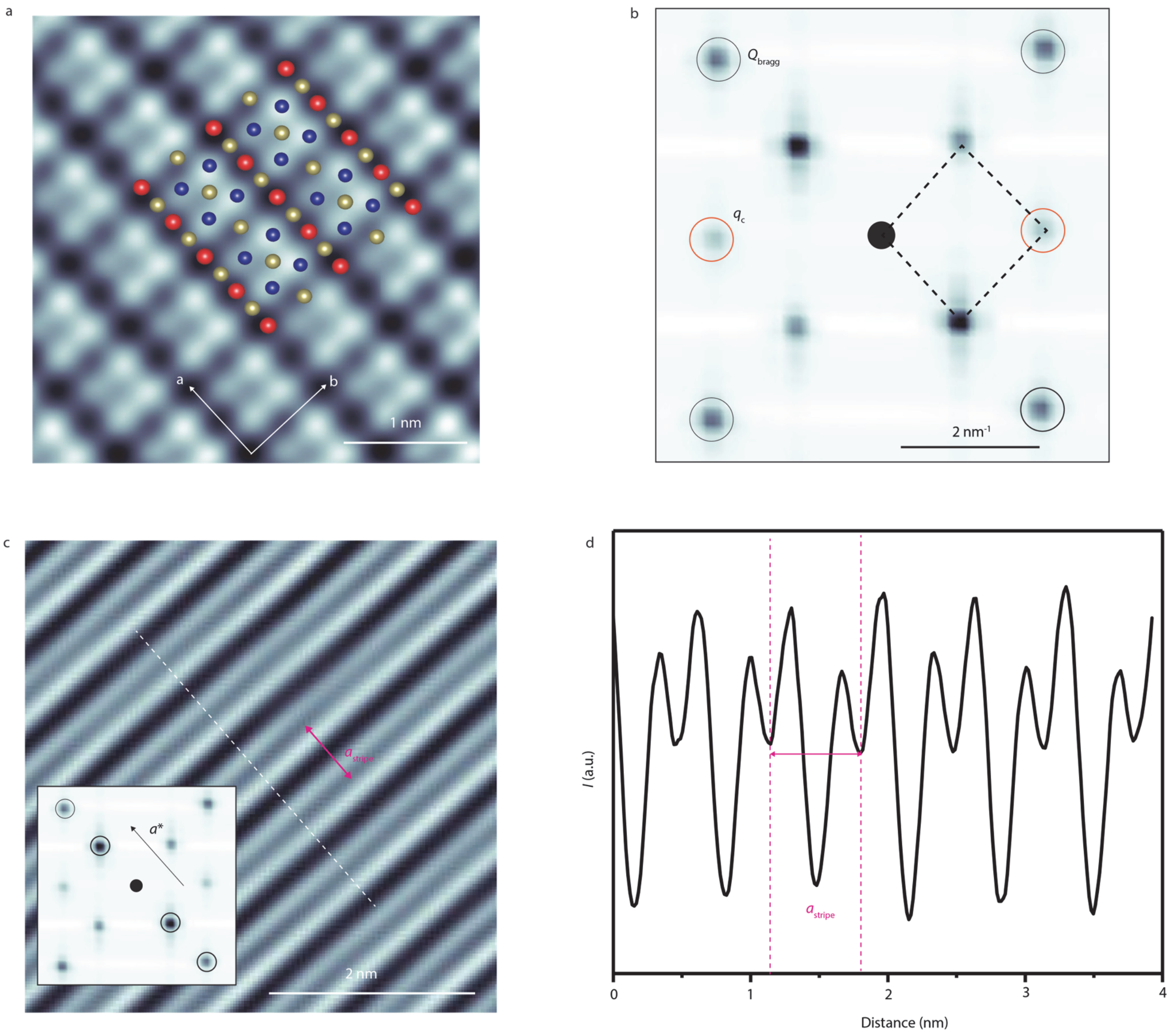

Figure S6. (**a**) High-resolution STM topographic image of $TaCo_2Te_2$ (taken at $T \sim 4.2$ K) with the atomic structure of the $TaCo_2Te_2$ top-layer superimposed. (**b**) FFT of the STM image in panel a, where Bragg and modulation peaks, marked with black and orange circles, respectively. (**c**) The real space pattern obtained by performing an inverse FFT on peaks along the a^* direction, as shown in the FFT inset image. Intensity profile of the stripe-like pattern was collected along the dashed white line. The real-space modulation vector is a_{stripe}. (**d**) Intensity line-cut to determine the real space modulation, a_{stripe}, of the stripe-like pattern shown in panel c. Reciprocal-space images are indexed relative to the RT-distorted structure.

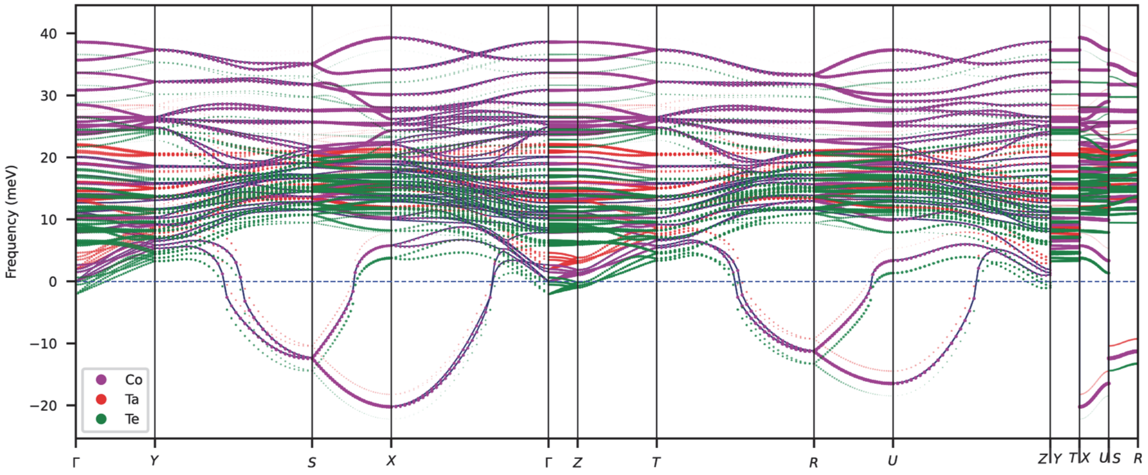

Figure S7. Phonon band calculations of the undistorted $TaCo_2Te_2$ with projections from each element. Phonon bands of each element are offset for clarity. Bands of all elements contribute to dynamic structural instabilities in the undistorted $TaCo_2Te_2$.

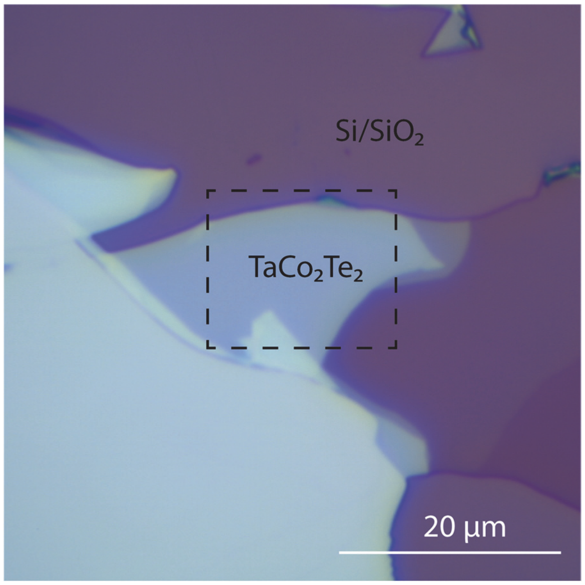

Figure S8. Optical microscope image of exfoliated $TaCo_2Te_2$ nanoflakes on a 285 nm SiO_2/Si substrate. The one outlined with a dashed black box is used for *in situ* heating Raman measurements.

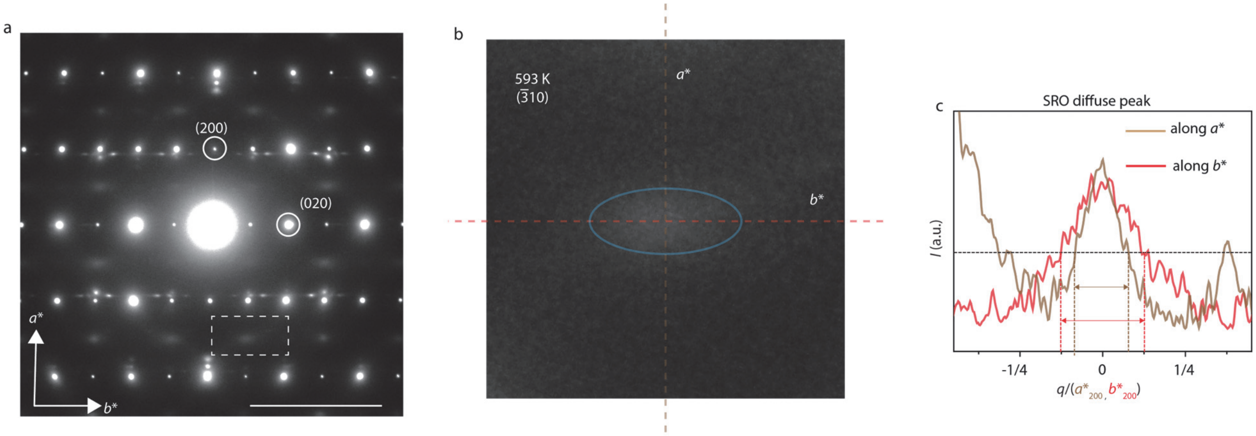

Figure S9. Correlation length of short-range order (SRO) above T_C in $TaCo_2Te_2$ determined from SAED images. (**a**) Representative SAED pattern at 593 K (> T_C), diffuse SRO peaks emerge after transitioning from the distorted $TaCo_2Te_2$ phase. (**b**) Intensity spread of a representative diffuse SRO peak in reciprocal space, marked with a white dashed box in panel a. (**c**) Intensity line scan of the diffuse SRO peak highlighted with a blue ellipsoid, obtained along *a** and *b** directions shown in panel b. Correlation length is determined at FWHM intensity. Scale bar in panel a is 5 nm^{-1}.

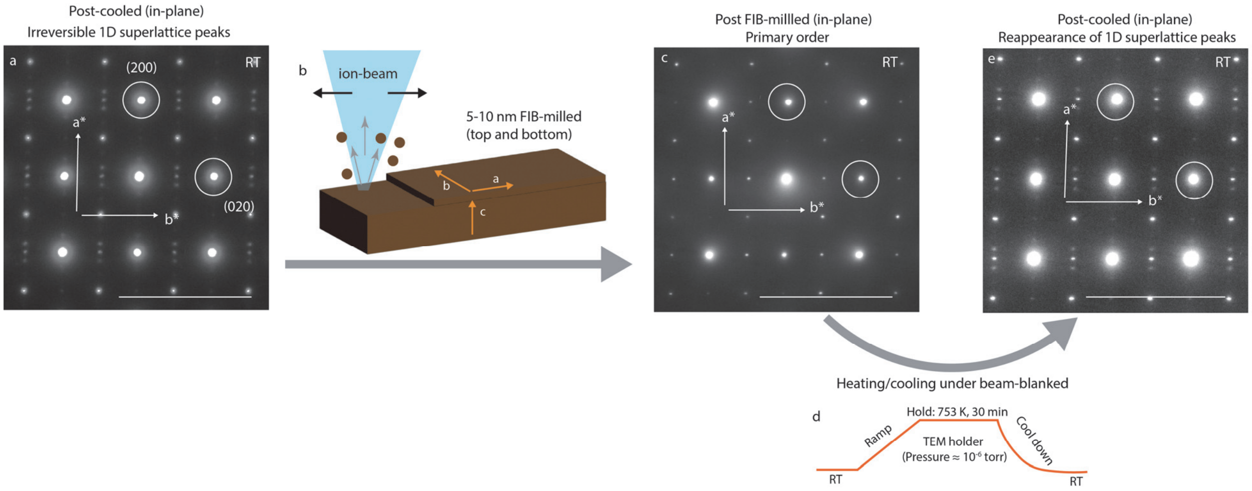

Figure S10. Superlattice peaks from the crystalline surface layer and $TaCo_2Te_2$ were determined from the FIB-prepared in-plane lamella. (**a**) RT SAED image of the post-cooled sample after annealing the sample at 753 K for 30 mins (above T_C), showing irreversible 1D superlattice peaks. (**b**) Schematic illustrating the FIB-milling near the top and bottom of the post-cooled sample shown in panel a (**c**) SAED image of the post-milled sample, showing no superlattice peaks and only the primary order ($TaCo_2Te_2$ distorted structure) remaining. **(d**) Complete heating cycle used in the TEM with an *in situ* heating holder under beam-blanked condition. (**e**) SAED image of the post-cooled sample under beam-blanked condition, showing reappearance of 1D superlattice peaks. Scale bar in panels a,c,and e is 5 nm^{-1}

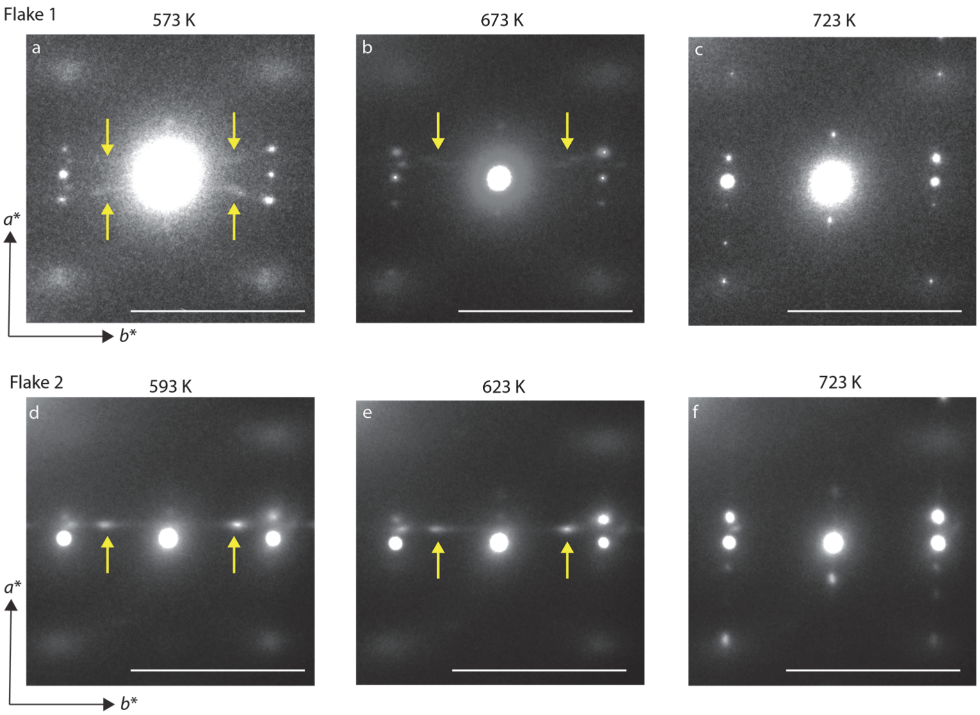

Figure S11. Temperature evolution of weakly coherent peaks above T_c. (**a-f**) SAED images of the *ab*-plane of two $TaCo_2Te_2$ exfoliated nanoflakes (Flake 1 and 2). Corresponding zoomed images of areas near the main-reflection peak of $TaCo_2Te_2$. As the temperature increases above T_c, the weakly coherent streaks (marked with yellow arrows) gradually disappear, while the intensity of the superlattice peaks along the a^* direction increases. Scale bar in panels a-f is 5 nm^{-1}.

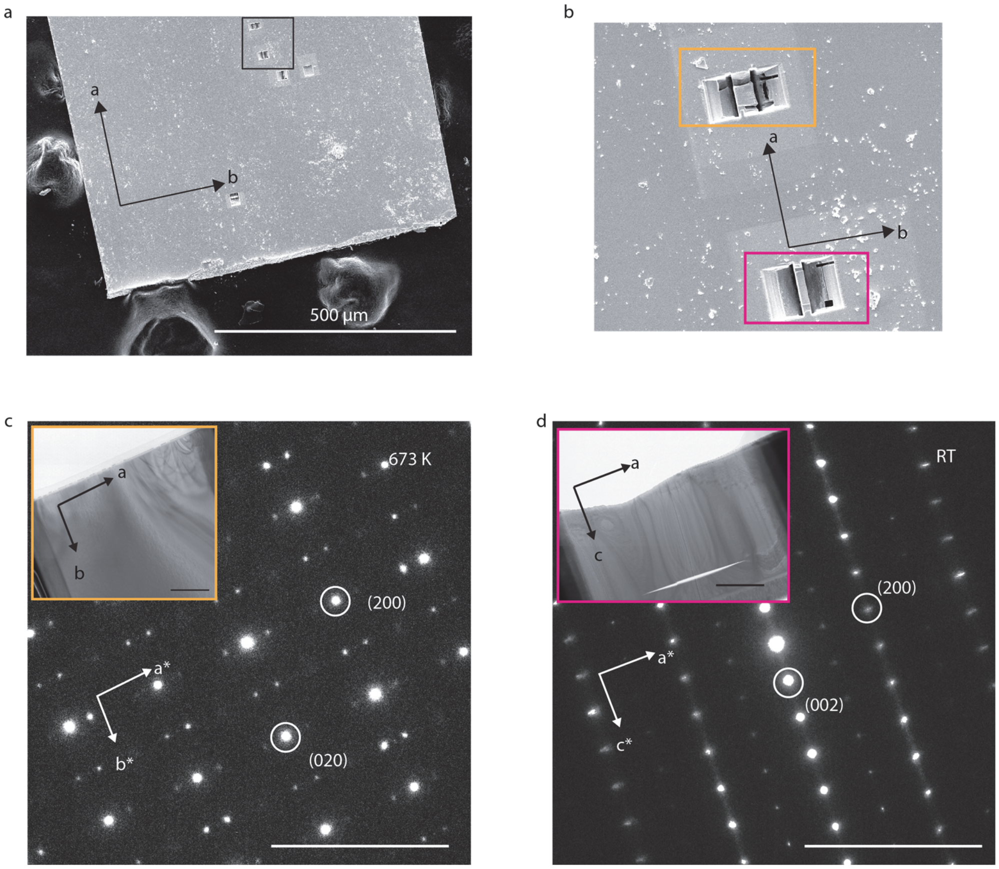

Figure S12. Orientation of superlattice structure. (**a**) SEM image of an oriented single crystal of $TaCo_2Te_2$ was used to cut the FIB lamella (**b**) Zoomed-in image of the two lamellas cut in in-plane (*ab*-plane) and cross-section (*ac*-plane), highlighted with orange and maroon boxes, respectively. (**c**) The SAED image of the in-plane sample collected at 673 K shows superlattice peaks along the *a** direction, which align with the bright-field TEM image of the FIB-cut lamella shown in the inset. (**d**) Similarly, the SAED image and the inset bright-field image of the FIB-cut lamella of the cross-section $TaCo_2Te_2$ of the *ac*-plane further confirm the direction of the superlattice peaks. Scale bar in panels c and d is 5 nm^{-1}. Scale bar for the inset images in panels c and d is 500 nm.

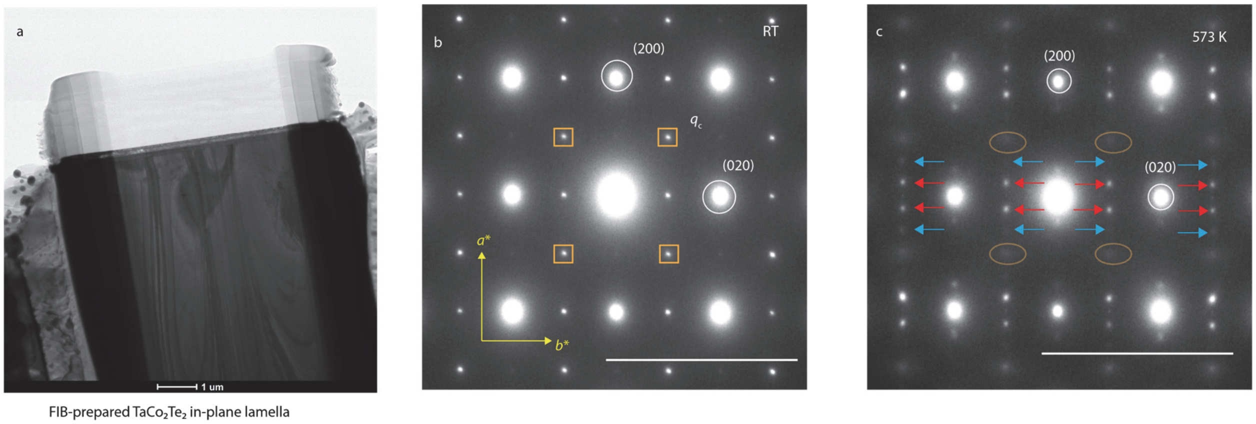

Figure S13. Emergence of 1D superlattice peaks in FIB-prepared in-plane lamella of $TaCo_2Te_2$. (**a**) Bright-field TEM image of the FIB-prepared $TaCo_2Te_2$. SAED patterns collected at (**b**) RT and (**c**) 573 K (above T_C). Orange ellipsoids show diffuse peaks at q_c. Red and blue arrows highlight the 1D superlattice peaks at q_1 and q_2, respectively, in panel c. Scale bar in panels b and c is 5 nm^{-1}.

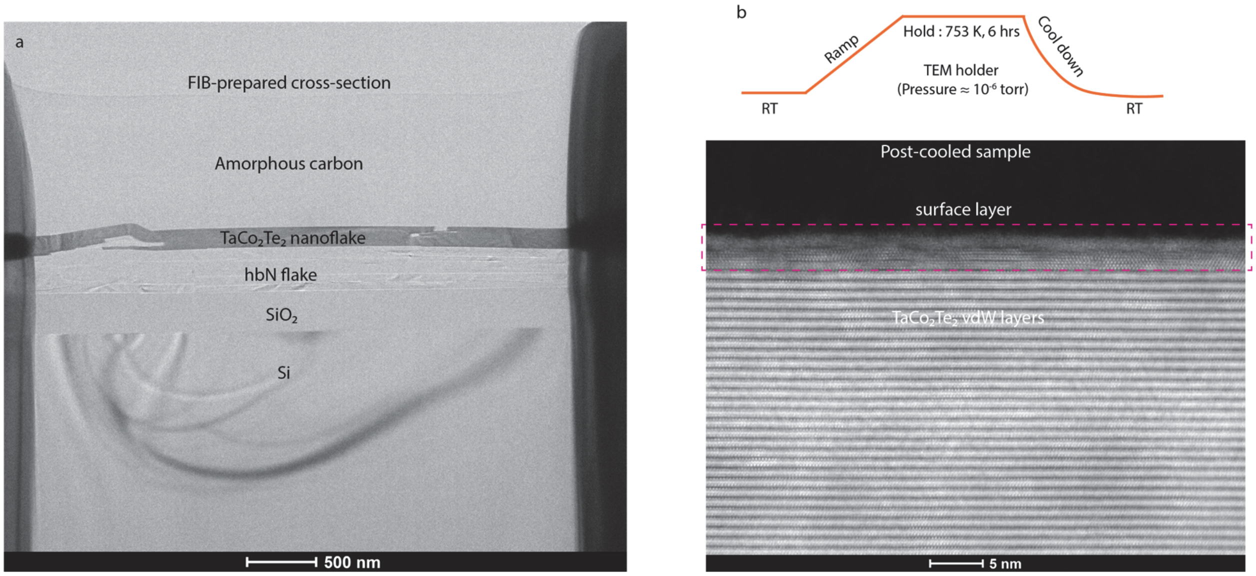

Figure S14. Cross-section FIB-prepared TEM sample to image the surface layer after heating above T_C. (**a**) Bright-field TEM image of a representative $TaCo_2Te_2$ cross-section sample prepared by FIB. The sample was prepared from an exfoliated $TaCo_2Te_2$ nanoflake, which was transferred onto a hBN exfoliated flake on a SiO_2/Si substrate. Before FIB milling, the sample was coated with amorphous carbon to prevent significant beam damage. (**b**) Complete heating cycle used in the TEM with an *in situ* heating holder on the sample shown in panel a, before STEM imaging. Low-magnification STEM image showing the area marked with a dashed pink box where a new surface layer is evident on top of $TaCo_2Te_2$ vdW layers. This is a low-magnification image of the one shown in Figure 5a in the main text.

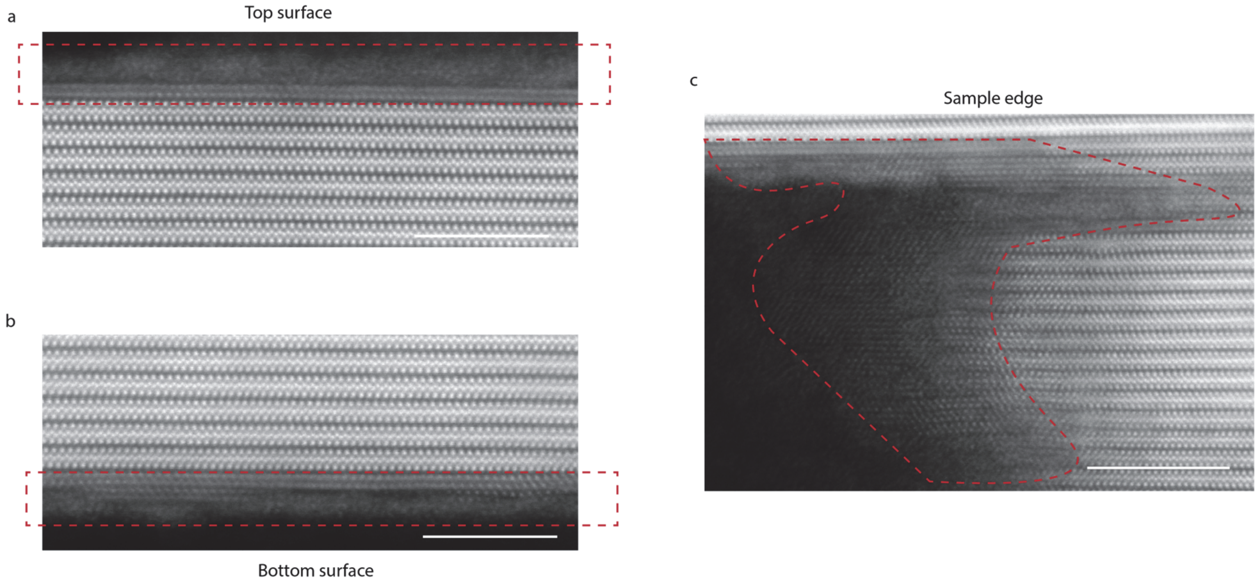

Figure S15. STEM images of the surface layer (ordered Co-Te and amorphous Ta-O) across the TEM sample imaged at (**a**) the top area, (**b**) the bottom area, and (**c**) near an unprotected sample edge. Red color dash outline highlights the surface layers. Scale bar in panels a-c is 5 nm.

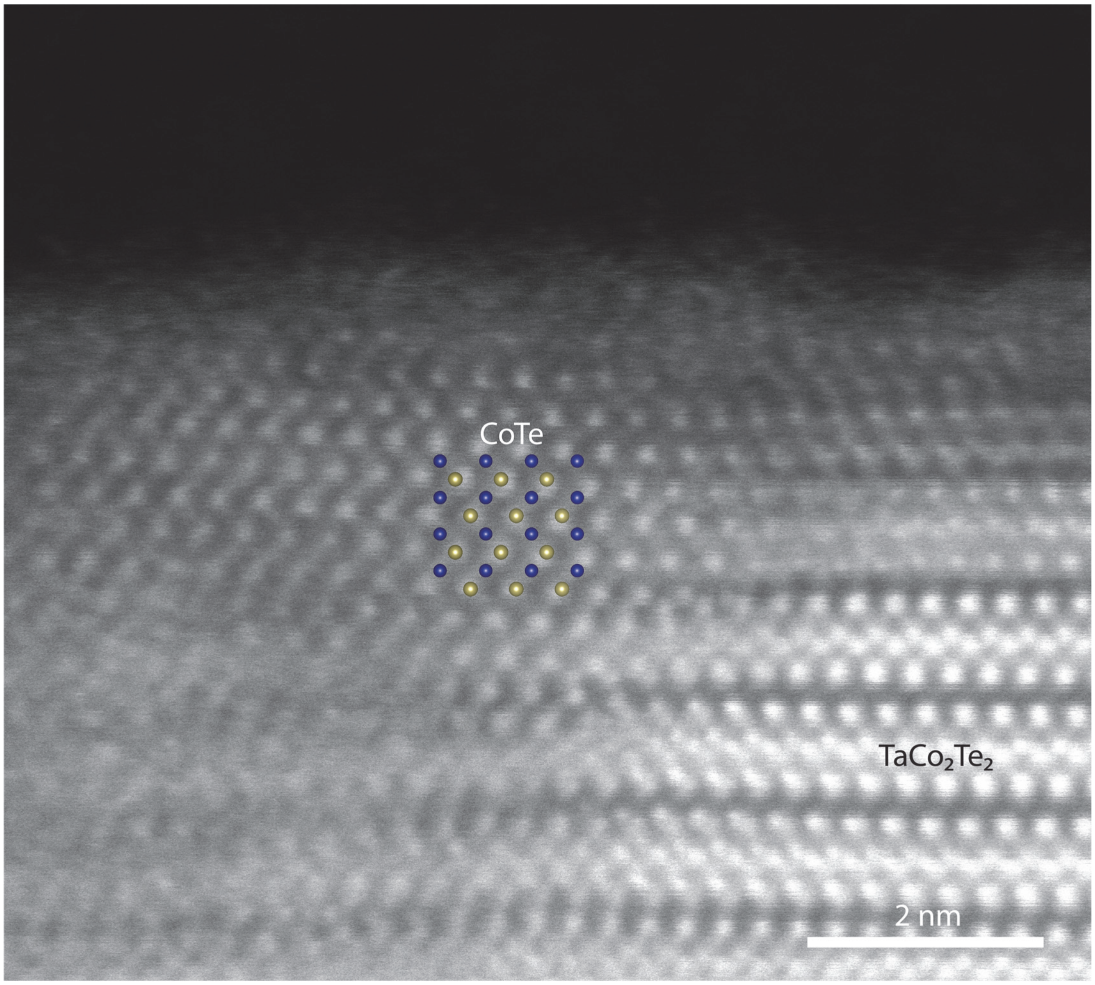

Figure S16. Cross-sectional atomic-resolution STEM image of the $TaCo_2Te_2$ nanoflake showing the ordered surface layer, which closely matches the 3D hexagonal CoTe structure.

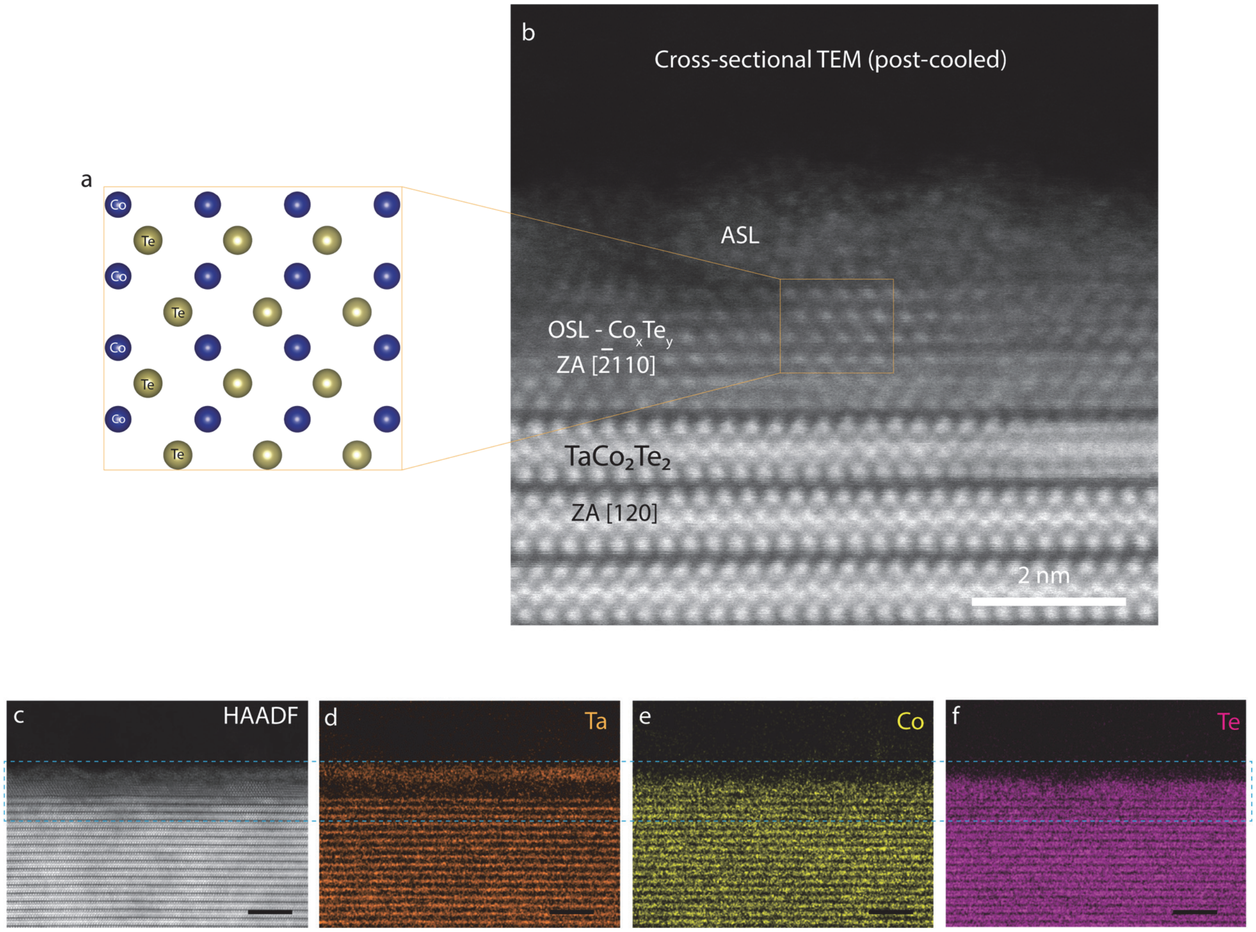

Figure S17. More cross-sectional TEM images for epilayer structural and compositional analysis. (**a,b**) Schematic showing the epilayer CoTe hexagonal structure (space group #194), matching the cross-sectional atomic-resolution ADF-STEM image of the Co_xTe_y ordered surface layer (OSL) near the surface of the $TaCo_2Te_2$ lamella. (**c-f**) Elemental mapping of the corresponding cross-sectional HAADF image. The blue dashed box highlights Ta migration in the amorphous surface layer (ASL) and Co-Te forms OSL. Scale bar in panels c-f is 5 nm.

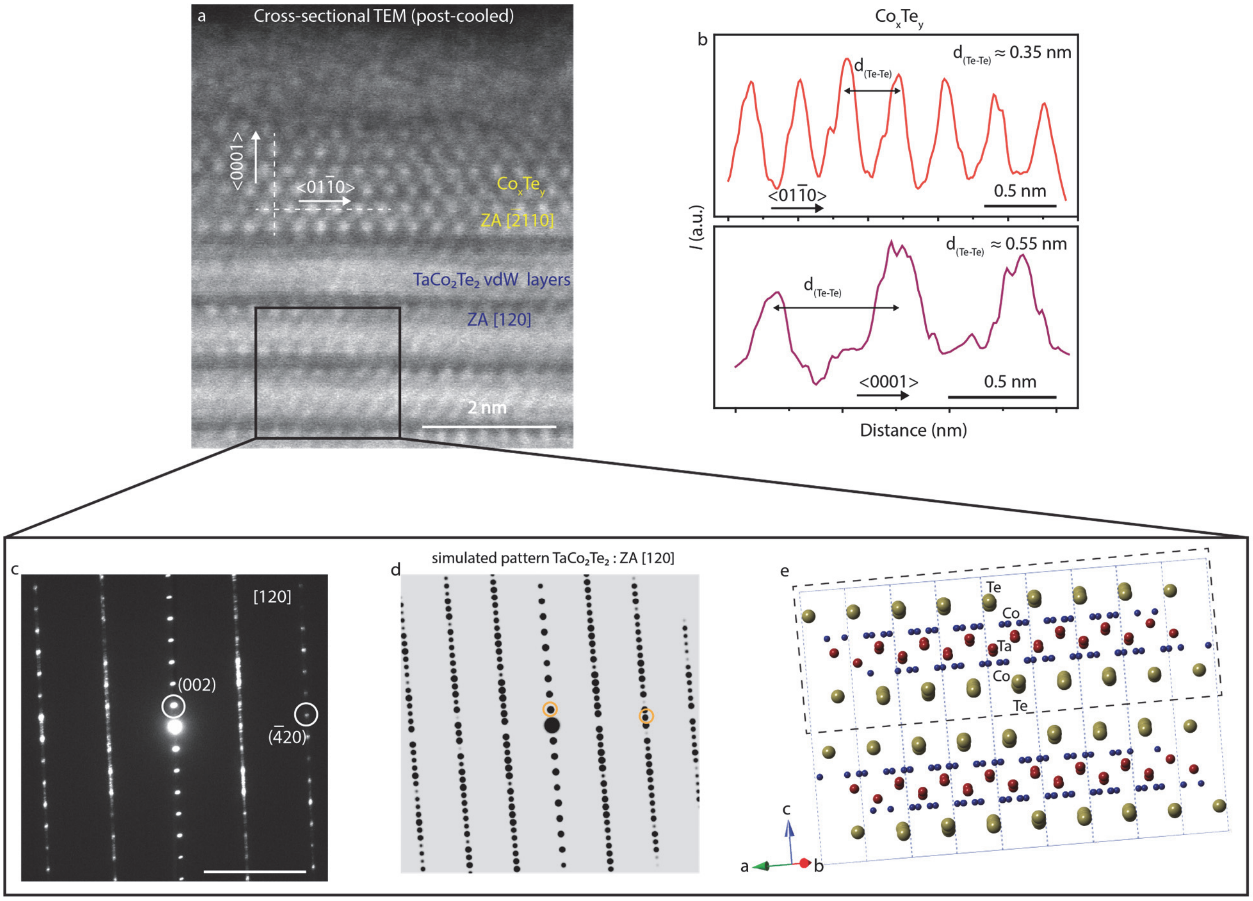

Figure S18. Crystallographic orientation of $Co_xTe_y/TaCo_2Te_2$ heterostructure. (**a**) Cross-sectional atomic resolution ADF-STEM image of the $Co_xTe_y/TaCo_2Te_2$ QvdW interface near the surface of the lamella. (**b**) Intensity line scan plots along the two perpendicular directions, marked with white dashed lines in panel a. (**c**) SAED pattern collected on the $TaCo_2Te_2$ cross-section matches with the (**d**) simulated pattern along the zone axis (ZA) of [120]. (**e**) The atomic arrangements of $TaCo_2Te_2$ along the [120] direction match the cross-sectional STEM images. Scale bar in panel c is 5 nm^{-1}.

Supplementary Note 3.

The cross-sectional STEM image displays an ordered surface layer interfaced with the $TaCo_2Te_2$ vdW substrate. The measured atomic distance between Te atoms (d_{Te-Te}) along the <0001> and <01−10> directions are approximately 0.55 nm and 0.35 nm, respectively. These values closely match the theoretical values of hexagonal CoTe (≈ 0.54 nm and ≈ 0.34 nm)

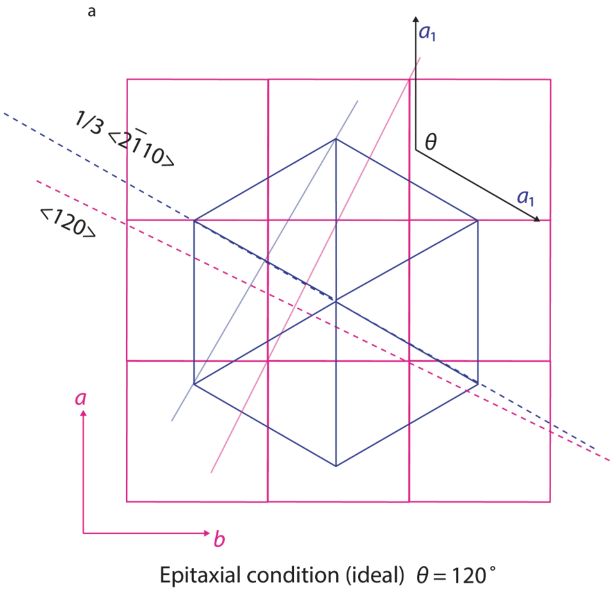

b

	along *a*	along *b*
Lattice mismatch (Δ)	$\frac{\lvert a_1 - a_{200} \rvert}{a_{200}}$	$\frac{\lvert a_1 \sin\theta - b_{020} \rvert}{b_{020}}$
Δ(%)	17.5%	2.28%
Moiré period (λ_{cal})	2.2 nm	14.7 nm
superlattice periodicity (λ_{exp})	2.72 nm (q_1)	—
$\frac{\lvert \lambda_{cal} - \lambda_{exp} \rvert}{\lambda_{cal}}$ (%)	23.6%	—

Figure S19. Lattice mismatch and Moiré periodicity are calculated using the ideal epitaxial condition between CoTe and $TaCo_2Te_2$. (**a**) Schematic illustrating the alignment of the CoTe epilayer with the $TaCo_2Te_2$ substrate. (**b**) Tabulated theoretical lattice mismatch (Δ) and corresponding Moiré periodicity, which are then compared with experimental data. The CoTe hexagonal (space group 194) and $TaCo_2Te_2$ (undistorted) lattice parameters are used for calculation.

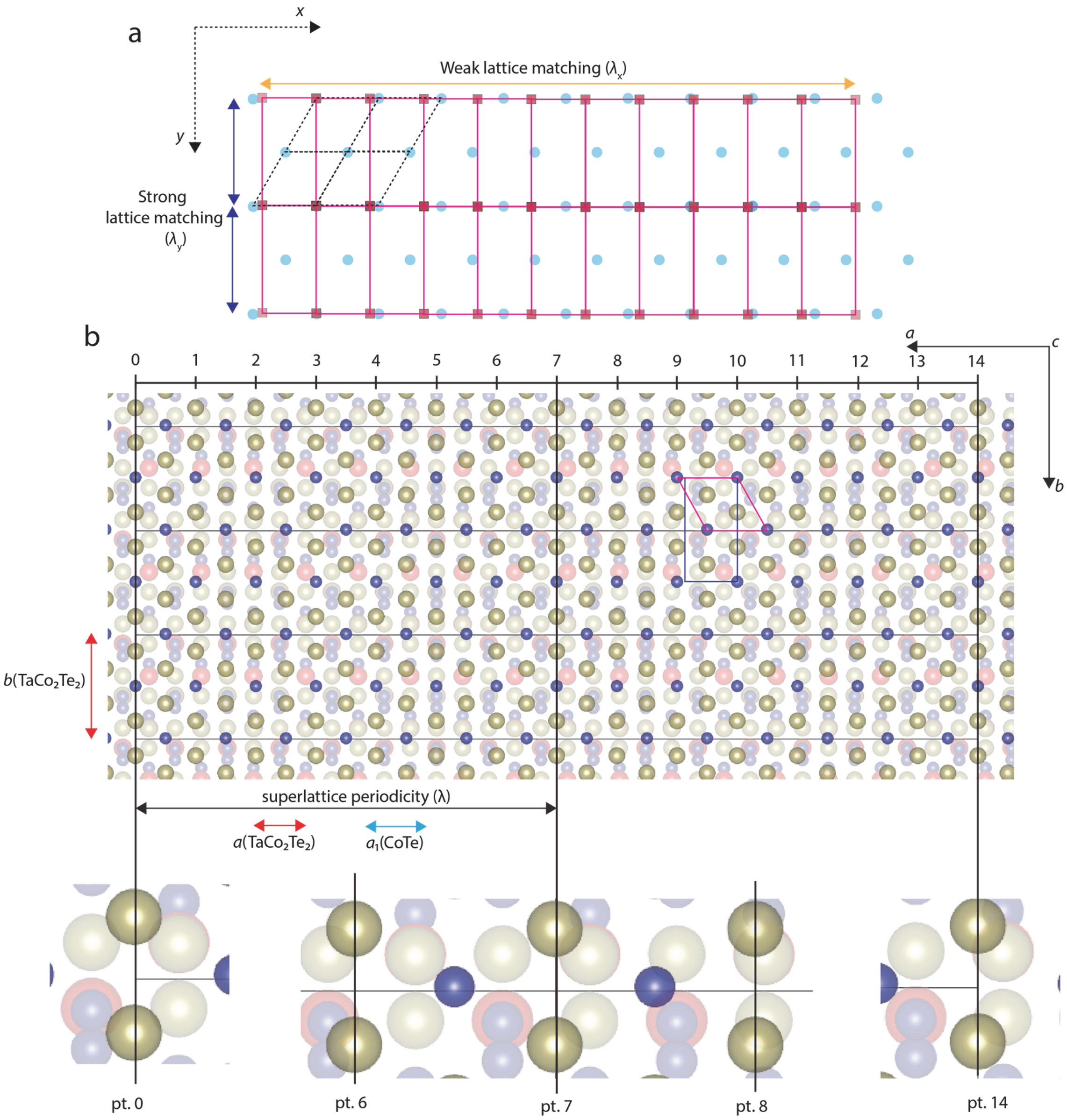

Figure S20: In-plane real space model of epitaxial $TaCo_2Te_2$/CoTe heterostructure. (**a**) Simplified model featuring overlaid unit cells of hexagonal CoTe and orthorhombic $TaCo_2Te_2$, illustrating strong lattice matching along the *y*-axis and weaker matching along the *x*-axis. (**b**) The atomic structure of the $TaCo_2Te_2$/CoTe heterointerface. The heterointerface is divided equally into a_1-length space points along the *a*-axis to examine overlapping lattice points. Zoom-in images for multiple lattice sites do not show clear signs of lattice commensuration along the *a*-axis. Unit cells of $TaCo_2Te_2$ (undistorted-orthorhombic) and CoTe (hexagonal) are marked with red and blue boxes, respectively.

Supplementary Note 4.

The coincidence site lattice (CSL) model repeats CSL sites at a distance (λ) along the *a*-axis to achieve lattice commensuration and a low-energy interface. Here is the condition for the lattice commensuration:

$$\lambda = ma_1 = na \qquad \text{Eq.1}$$

where a_1 and a are the lattice parameters of Co_xTe_y and undistorted $TaCo_2Te_2$, respectively, and λ is the length of the commensurate superlattice along the *a*-axis.

Here, we are assuming that the Co_xTe_y forms a hexagonal unit cell without any structural modulations. The heterostructure shows strong lattice matching along the *b*-axis, where d_{010} (CoTe) ≈ d_{020} ($TaCo_2Te_2$), and d_{hkl} is the interplanar distance. Using this lattice vector relationship, we obtain:

$$\left(\sqrt{3}/2\right) a_1 \approx b/2 \qquad \text{Eq.2}$$

Now, substituting a_1 in Eq.1 using Eq.2, we get:

$$\frac{m}{n} \approx \sqrt{3}\,\frac{a}{b}(T) \quad , T \text{ is temperature} \qquad \text{Eq.3}$$

Here, we assume that the thermal expansion of $TaCo_2Te_2$ is isotropic and calculate $m/n \approx 0.869$.

We then use the experimental values for the modulation length of the q_1 and q_2, and obtain non-integer values of $(m, n) \approx (7.18, 8.26)$ for q_1 and (2.94, 3.38) for q_2, indicating a clear deviation from a commensurate superlattice along the *a*-axis.

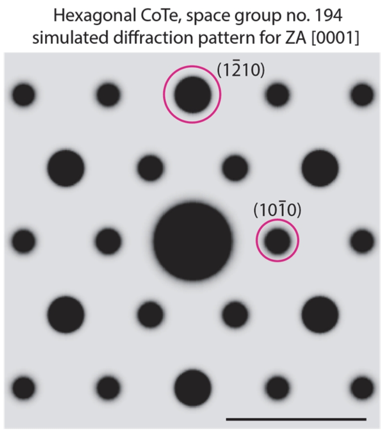

Figure S21. Simulated diffraction pattern of hexagonal CoTe along zone axis [0001]. Scale bar is 5 nm^{-1}.

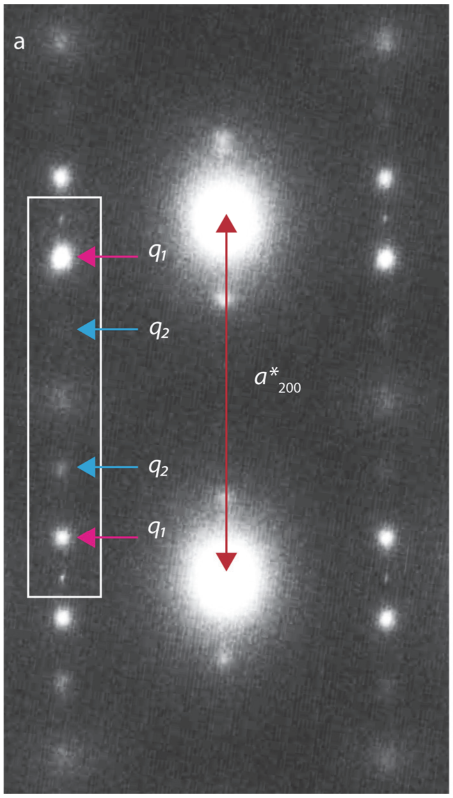

b

Nanoflake	Preparation	Temperature (K)	q_1 ($*a_{200}$)	q_2 ($*a_{200}$)
1	Exfoliation	723	0.121	0.291
2	Exfoliation	723	0.125	0.292
3	Exfoliation	723	0.126	0.296
4	FIB	723	0.117	0.296
5	FIB	753	0.116	0.299

Figure S22. Determining length of modulation wave vectors from SAED images: (**a**) Representative SAED image marked with incommensurate wave vectors (q_1, q_2) along the a^* direction, measured with respect to a^*_{200} (**b**) Tabulated reciprocal wavevector distances of q_1 and q_2 from various samples prepared either by FIB or exfoliation methods at T > 700 K.

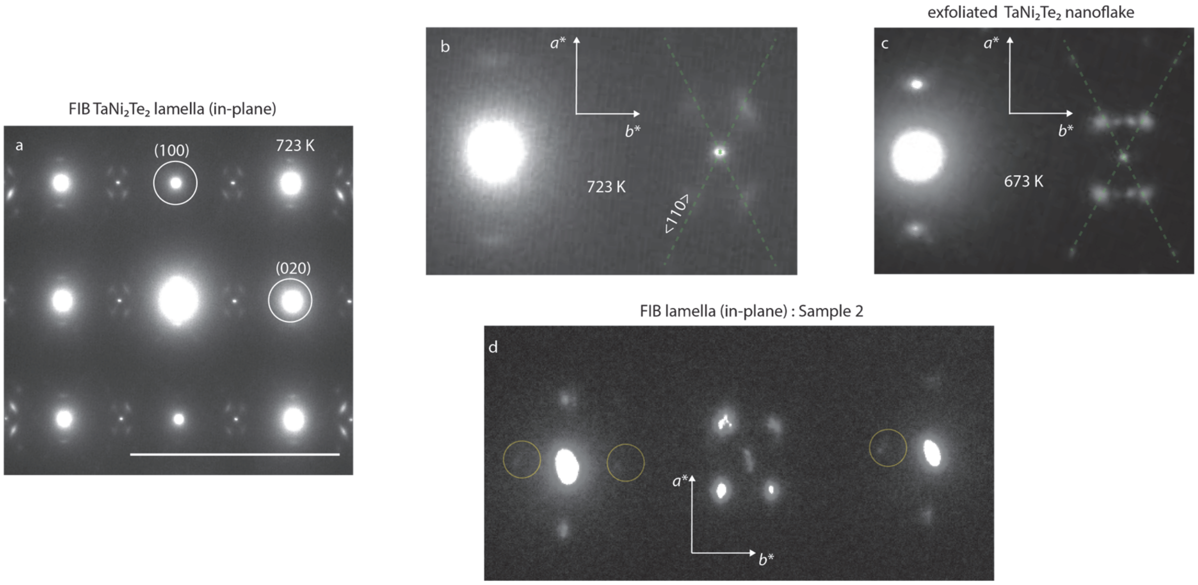

Figure S23. Comparing SAED images of $TaNi_2Te_2$ samples (**a**) In-plane SAED image of FIB-prepared $TaNi_2Te_2$ sample at 723 K with main reflections and diffuse superlattice peaks. (**b**) Zoom-in image of the diffuse and twinned superlattice peaks shown in panel a (**c**). In comparison, the $TaNi_2Te_2$ exfoliated flake shows more coherence at 673 K. (**d**) Another FIB-prepared $TaNi_2Te_2$ sample shows new superlattice peaks emerging along the *b** direction, not observed in panels b and c. Scale bar in panel a is 5 nm^{-1}.

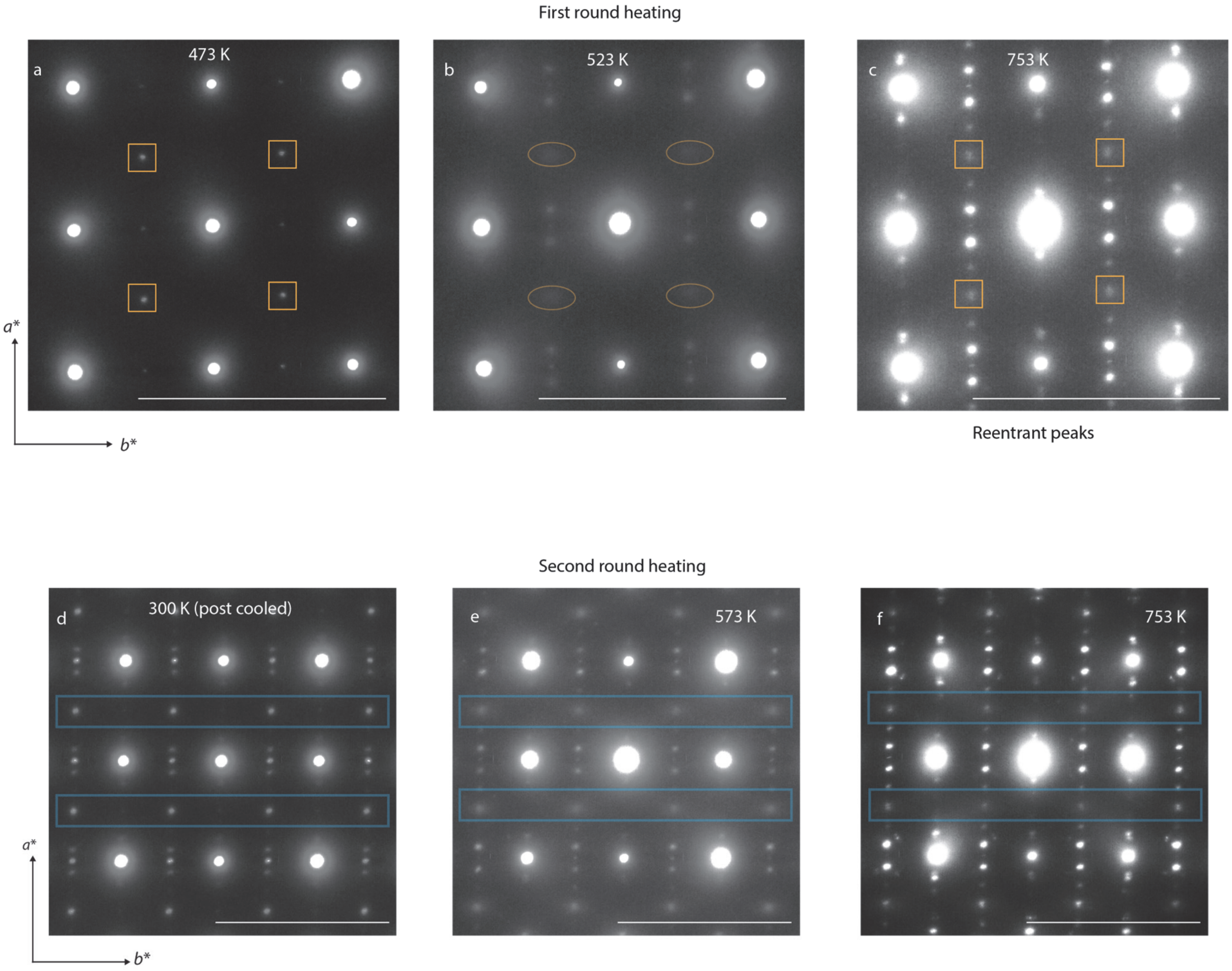

Figure S24. Reentrant distorted peaks above T_C in $TaCo_2Te_2$ with another sample. (**a-c**) During the first-round heating, the coherent q_c peak at 473 K below T_C becomes diffuse near T_C, around 523 K, and then regains coherence at 753 K as a reentrant phase. (**d-f**) During the second-round heating of the post-cooled sample, the reentrant phase persists. In panels a-c, coherent q_c peaks are marked with orange boxes and diffuse q_c peaks are marked with orange ellipsoids. In panel d-f, blue boxes highlight the q_c peaks. Scale bar in all panels is $5nm^{-1}$.

.

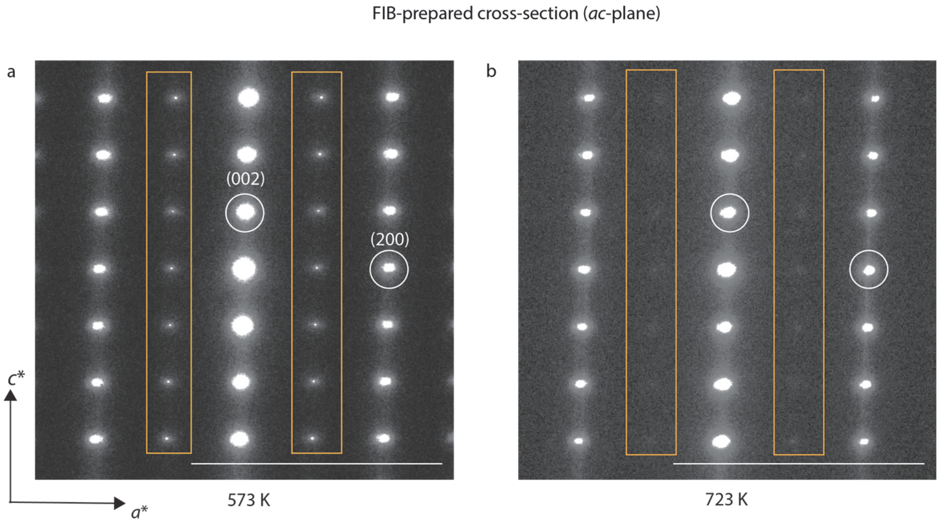

Figure S25. SAED images of a FIB-prepared cross-section of *ac*-plane collected at (**a**) 573 K and (**b**) 723 K. Orange boxes highlight coherent q_c peaks at 573 K, which almost disappear completely at 723 K. No coherent superlattice peaks appear along *a**direction. Scale bar in panels a,b is 5 nm^{-1}.

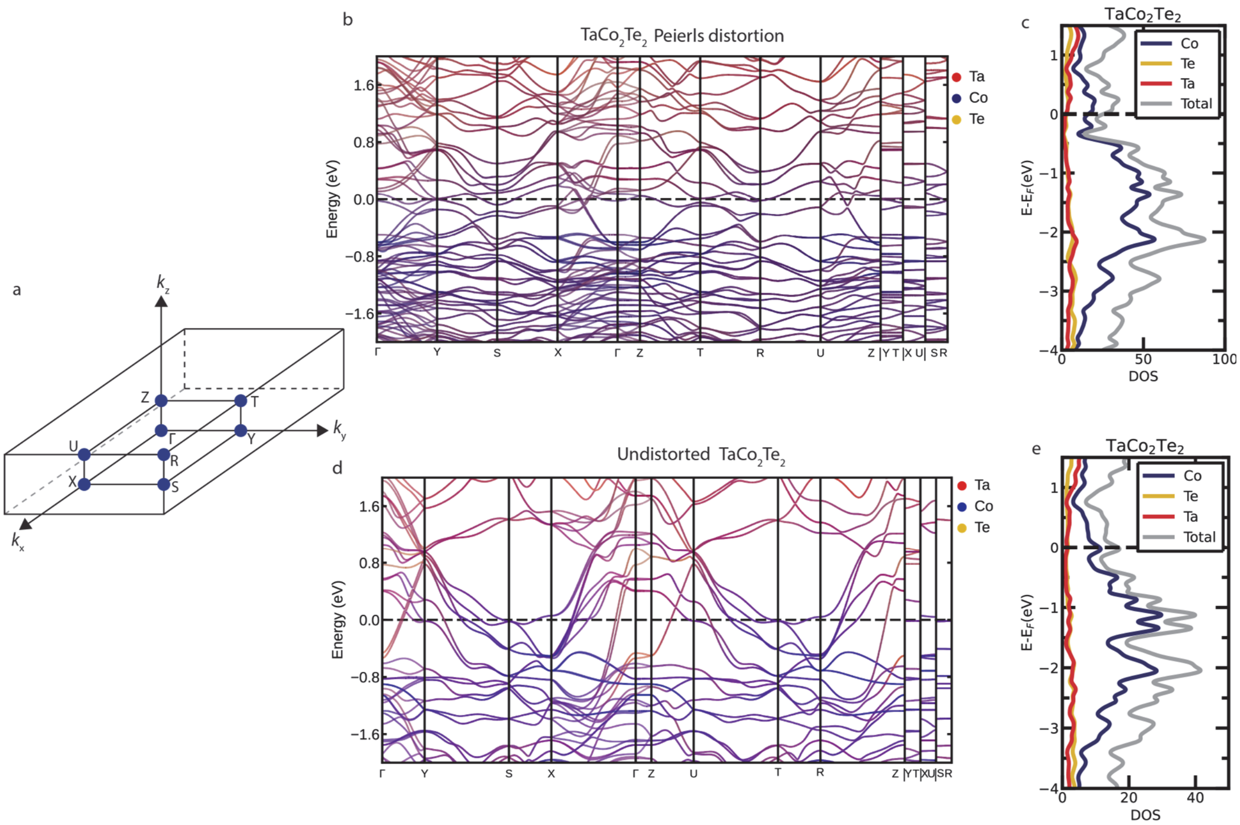

Figure S26. Calculated electronic band-structures of $TaCo_2Te_2$**.** (**a**) 3D Brillouin zone of the $TaCo_2Te_2$ structure used for first-principles calculations. (**b**) Electronic band-structure of Peierls distorted $TaCo_2Te_2$ with (**c**) corresponding density of states (DOS) over a large energy range. (**d**) Electronic band-structure of undistorted $TaCo_2Te_2$ with (**e**) corresponding DOS over a large energy range. Both structures show metallic bands near the Fermi energy (E_F).

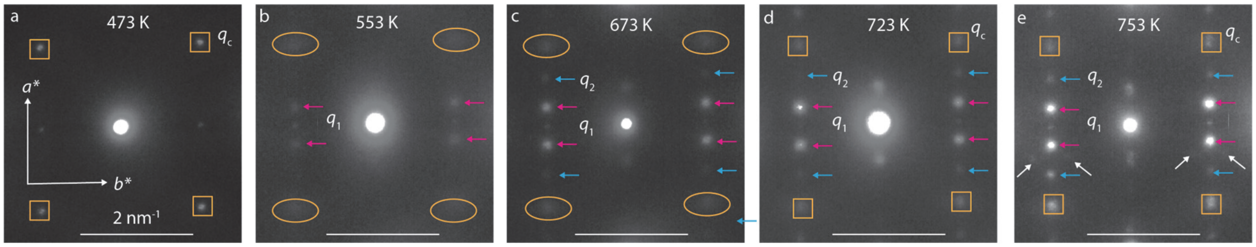

Figure S27. (**a-e**)Temperature-dependent evolution of structural modulation at the $Co_xTe_y/TaCo_2Te_2$ heterointerface evaluated with SAED patterns collected *via in situ* heating in a TEM. In panel e, white arrows correspond to coherent peaks deviating from the *a** direction.
